# Vitamin C for Preventing and Treating the Common Cold

**DOI:** 10.1371/journal.pmed.0020168

**Published:** 2005-06-28

**Authors:** Robert M Douglas, Harri Hemilä

## Abstract

Whether vitamin C has an effect on the common cold has been a subject of controversy for at least 60 years. What does the evidence show?

The role of vitamin C in the prevention and treatment of the common cold has been a subject of controversy for at least 60 years. Public interest in the subject, stimulated originally by the vigorous advocacy of Nobel laureate Linus Pauling during the 1970s [1], continues to be high. We have recently updated the Cochrane Review [2] on this topic (Text S1), incorporating 55 comparative studies that have been carried out over a period of 65 years.

## The Updated Review

**Figure 1:**
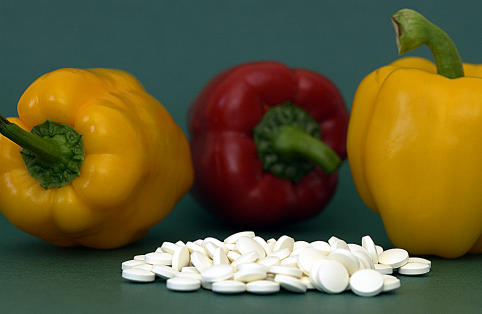
500-mg Vitamin C Tablets and Paprikas Vitamin C was identified in the 1930s by Albert Szent-Györgyi, who received his Nobel Prize partly for this work. He found that paprika is a particularly rich source of the vitamin, which made it possible to produce kilograms of it for research purposes ([1963] Annu Rev Biochem 32: 1–14). Nowadays, the most convenient way to increase vitamin C intake is by way of 500-mg tablets, but further research is needed to explore the conditions in which supplementation may be beneficial.

We sought to discover whether vitamin C in doses of 200 mg or more daily (Figure 1) reduces the incidence, duration, or severity of the common cold when used either as continuous prophylaxis or after the onset of cold symptoms. Criteria for inclusion were placebo-controlled trials to prevent or treat the common cold using oral doses of vitamin C of 200 mg/day or more. Literature from 1940 to 2004 was methodically screened.

An overview of the results of the three meta-analyses is shown in Table 1. Incidence was not altered in the subgroup of 23 community studies where prophylactic doses as high as 2 g daily were used. But a subgroup of six studies of marathon runners, skiers, and soldiers exposed to significant cold and/or physical stress experienced, on average, 50% reduction in common cold incidence.

**Table 1:**
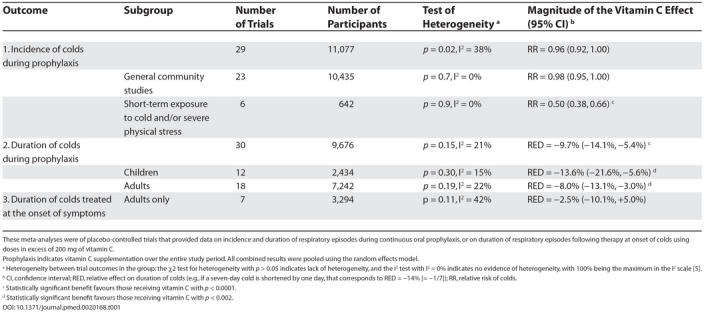
Results of the Three Meta-Analyses

These meta-analyses were of placebo-controlled trials that provided data on incidence and duration of respiratory episodes during continuous oral prophylaxis, or on duration of respiratory episodes following therapy at onset of colds using doses in excess of 200 mg of vitamin C.

Prophylaxis indicates vitamin C supplementation over the entire study period. All combined results were pooled using the random effects model.

^a^ Heterogeneity between trial outcomes in the group: the 2 test for heterogeneity with *p* > 0.05 indicates lack of heterogeneity, and the I^2^ test with I^2^ = 0% indicates no evidence of heterogeneity, with 100% being the maximum in the I^2^ scale [5].

^b^ CI, confidence interval; RED, relative effect on duration of colds (e.g., if a seven-day cold is shortened by one day, that corresponds to RED = -14% [= -1/7]); RR, relative risk of colds.

^c^ Statistically significant benefit favours those receiving vitamin C with *p* < 0.0001.

^d^ Statistically significant benefit favours those receiving vitamin C with *p* < 0.002.

Duration of cold episodes that occurred during prophylaxis was significantly reduced in both children and adults. For children this represented an average reduction of 14% in symptom days, while in adults the reduction was 8%.

For the seven trials that evaluated the therapeutic impact of vitamin C used at the onset of symptoms (all in adults), benefits were not observed for duration of episodes, although one of the large trials recorded a statistically significant reduction in the duration of colds among participants administered a single vitamin C dose of 8 g on the day of symptom onset [3].

## Implications of the Review

The lack of effect of prophylactic vitamin C supplementation on the incidence of common cold in normal populations throws doubt on the utility of this wide practice. The clinical significance of the minor reduction in duration of common cold episodes experienced during prophylaxis is questionable, although the consistency of these findings points to a genuine biological effect.

In special circumstances, where people used prophylaxis prior to extreme physical exertion and/or exposure to significant cold stress, the collective evidence indicates that vitamin C supplementation may have a considerable beneficial effect; it was the results of one of these six trials, with schoolchildren in a skiing school [4], that particularly impressed Pauling [1]. However, great caution should be exercised in generalizing from this finding, which is based mainly on marathon runners.

No benefits have been observed from therapeutic use of doses totalling 10 g that was divided for the first three days of illness. The equivocal findings of the large study, which used 8 g only on the day of onset of respiratory symptoms [3], are tantalising and deserve further assessment.

None of the therapeutic trials carried out so far has examined the effect of vitamin C on children, even though the prophylaxis trials have shown substantially greater effect on episode duration in children.

Study quality for the trials included in these three meta-analyses was variable, but sensitivity analysis, where we excluded studies from the analysis that were less adequately blinded or randomized, did not change the general conclusions of the Cochrane Review.

Future work on this topic should explore the value of high dose therapy—in particular, in children—and the mechanisms underlying the observed prophylaxis benefits in those exposed to substantial physical and/or cold stress.

## Supporting Information

Text S1 Updated Cochrane Review Douglas RM, Hemilä H, D'Souza R, Chalker EB, Treacy B (2004) Vitamin C for preventing and treating the common cold. Cochrane Database Syst Rev 4: CD000980.pub2.
Date of most recent substantive amendment: 10 August 2004.
This data supplement can be freely accessed on the *PLoS Medicine* Web site, but it is not published under the Creative Commons Attribution License.
Copyright © 2004 The Cochrane Collaboration. Published by John Wiley and Sons. All rights reserved.
(1.3 MB PDF).
