# Supplementary material for: Vitamin C for Preventing and Treating the Common Cold
Source: PLoS Med. 2005 Jun 28;2(6):e168. doi: 10.1371/journal.pmed.0020168 (PMC1160577; doi:10.1371/journal.pmed.0020168)
Supplement: Text S1 — Douglas RM, Hemilä H, D'Souza R, Chalker EB, Treacy B (2004) Vitamin C for preventing and treating the common cold. Cochrane Database Syst Rev 4: CD000980.pub2. Date of most recent substantive amendment: 10 August 2004. This data supplement can be freely accessed on the PLoS Medicine Web site, but it is not published under the Creative Commons Attribution License. Copyright © 2004 The Cochrane Collaboration. Published by John Wiley and Sons. All rights reserved. (1.3 MB PDF). [file pmed.0020168.sd001.pdf]

VITAMIN C FOR PREVENTING AND TREATING THE COMMON COLD

Douglas RM, Hemila H, D'Souza R, Chalker EB, Treacy B

Date of most recent amendment: 06 July 2004
Date of most recent substantive amendment: 10 August 2004

This record should be cited as: Douglas RM, Hemila H, D'Souza R, Chalker EB, Treacy B. Vitamin C for preventing and treating the common cold. *The Cochrane Database of Systematic Reviews* 2004, Issue 4. Art. No.: CD000980.pub2. DOI: 10.1002/14651858.CD000980.pub2.

Copyright © 2004 The Cochrane Collaboration. Published by John Wiley & Sons, Ltd.
All rights reserved. No part of The Cochrane Library may be reproduced, stored in a retrieval system or transmitted in any form or by any means, electronic, mechanical, photocopying, recording, scanning or otherwise, except under the terms of the Copyright, Designs and Patents Act 1988 or under the terms of a licence issued by the Copyright Licensing Agency Ltd, 90 Tottenham Court Road, London W1T 4LP, UK, without the permission in writing of John Wiley & Sons, Ltd, The Atrium, Southern Gate, Chichester, West Sussex PO19 8SQ, UK, or emailed to permreq@wiley.co.uk, or faxed to (+44) 1243 770620.

ABSTRACT

Background

The role of oral vitamin C (ascorbic acid) in the prevention and treatment of the common cold has been a subject of controversy for at least sixty years. Public interest in the topic continues to be high and vitamin C continues to be widely sold and used as a preventive and therapeutic agent for this common ailment.

Objectives

To discover whether oral vitamin C in doses of 200 mg or more daily, reduces the incidence, duration or severity of the common cold when used either as continuous prophylaxis or after the onset of cold symptoms.

Search Strategy

This updated review added to earlier searches, a full search of the following electronic databases: the Central Register of Controlled Trials (CENTRAL) (*The Cochrane Library* Issue 2, 2004); MEDLINE (January 1966 to June 2004); and EMBASE (1990 to June 2004).

Selection Criteria

Papers were excluded if a dose less than 200 mg daily of vitamin C was used; if there was no placebo comparison; if methods of outcome assessment were inadequately described; and if the report did not record any of the three study outcomes (incidence, duration or severity) in sufficient detail to enter into the meta-analysis. Three criteria of study quality were assessed: Jadad scores, placebo distinguish-ability, and allocation concealment.

Data collection and analysis

Two reviewers independently extracted data and assessed trial quality. 'Incidence' of colds during prophylaxis was assessed as the proportion of participants experiencing one or more colds during the study period. 'Duration' was the mean days of illness of cold episodes and 'severity' of these episodes was assessed by days confined indoors, off work or school. or by symptom severity scores.

Main Results

Twenty-nine trial comparisons involving 11,077 study participants contributed to the meta-analysis on the relative risk (RR) of developing a cold while taking prophylaxis. The pooled RR was 0.96 (95% CI 0.92 to 1.00). A subgroup of six trials that involved a total of 642 marathon runners, skiers, and soldiers on sub-arctic exercises reported a pooled RR of 0.50 (95%CI 0.38 to 0.66).

Thirty comparisons that involved 9,676 respiratory episodes contributed to the meta-analysis on common cold duration during prophylaxis . A consistent benefit was observed, representing a reduction in cold duration of 8% (95% CI 3% to 13%) for adult participants and 13.5% (95% CI 5% to 21%) for child participants.

Fifteen trial comparisons that involved 7,045 respiratory episodes contributed to the meta-analysis of severity of episodes experienced while on prophylaxis. The pooled results revealed a difference favouring those on vitamin C when days confined to home and off work or school were taken as a measure of severity (p = 0.02), and when restricting to studies which used symptom severity scores (p = 0.16), and for the both measures of severity combined (p = 0.004).

Seven trial comparisons that involved 3,294 respiratory episodes contributed to the meta-analysis of cold duration during therapy with vitamin C that was initiated after the onset of cold symptoms, and no significant difference from placebo was seen.

Four trial comparisons that involved 2,753 respiratory episodes, contributed to the meta-analysis of cold severity during therapy and no significant difference from placebo was seen.

In laboratory studies, differing methods of artificial transmission of virus to vitamin C or placebo treated volunteers in residential experiments gave different results. Volunteers infected by nasal installation showed small or no benefit from vitamin C, whereas a group who were infected more naturally, reported less severe symptom severity scores (p = 0.04).

Reviewers' conclusions

The failure of vitamin C supplementation to reduce the incidence of colds in the normal population indicates that routine mega-dose prophylaxis is not rationally justified for community use. But evidence shows that it could be justified in persons exposed to brief periods of severe physical exercise and/or cold environments. Also, the consistent and statistically significant small benefits on duration and severity for those using regular vitamin C prophylaxis indicates that vitamin C plays some role in respiratory defence mechanisms. The trials in which vitamin C was introduced at the onset of colds as therapy did not show any benefit in doses up to 4 grams daily, but one large trial reported equivocal benefit from an 8 gram therapeutic dose at onset of symptoms.

This record should be cited as:
Douglas RM, Hemila H, D'Souza R, Chalker EB, Treacy B. Vitamin C for preventing and treating the common cold. *The Cochrane Database of Systematic Reviews* 2004, Issue 4. Art. No.: CD000980.pub2. DOI: 10.1002/14651858.CD000980.pub2.

BACKGROUND

Numerous animal studies with different species have shown that vitamin C affects resistance to diverse infections by viruses and bacteria (Hemilä 1997c) . It might therefore be expected that this vitamin would also play such a role in human beings, but its importance in this regard is unresolved. Since the early 1940s, a large number of controlled trials have been carried out to examine the possible effects of vitamin C on the common cold, a ubiquitous problem caused by a wide range of viral agents. The common cold causes enormous morbidity

worldwide and the search for simple and effective preventive and/or therapeutic agents has been elusive.

In 1970, the publication of Pauling 1970a, a book for the general public entitled "Vitamin C and the Common Cold" generated huge public interest which persists today. Linus Pauling was a double Nobel Laureate in chemistry and peace. In Pauling 1971a he carried out a meta-analysis in which he combined the p-values derived from four placebo-controlled trials by Fisher's method and found that there was strong evidence that vitamin C decreases the 'incidence of colds' (p = 0.003). In a second meta-analysis, Pauling 1971b focused on 'days of illness per person' in the best of these four trials Cowan 1942, Ritzel 1961 and combining the p-values by Fisher's method led him to conclude that "the null hypothesis of equal effectiveness of ascorbic acid and placebo is rejected at the level p less than 0.001."

Ritzel 1961 had reported a brief randomised controlled trial of children at a ski school in the Swiss Alps in which he administered 1 g daily and found reduced incidence and duration of colds in the recipients of vitamin C. Pauling put much weight on the Ritzel trial and based his expectations of vitamin C benefits on it. Pauling 1970b and Pauling 1976 also presented other data suggesting that human diets might not provide sufficient intake of vitamin C for optimal health, and proposed that mega-dose supplementation might profoundly influence both the incidence and severity of the common cold.

Pauling's advocacy of vitamin C led to numerous careful trials in a number of countries in the following decade, the largest of which were performed on healthy adult volunteers in Canada (Anderson 1972; Anderson 1974a; Anderson 1975a).

The evidence emerging from these trials was often confusing (Anderson 1977), but generally failed to support Pauling's hope that vitamin C would be a panacea. Chalmers 1975 calculated an unweighted average of the treatment effect in seven placebo-controlled trials and found that colds in vitamin C groups were 0.11 ± 0.24 standard error (SE) days shorter, and the incidence of colds in vitamin C groups was 0.09 ± 0.06 (SE) episodes less per year, neither of which is a statistically or clinically significant difference. In a qualitative review on vitamin C and the common cold published in the same year, Dykes 1975 also concluded that vitamin C had no effects on colds.

The reviews by Chalmers 1975 and Dykes 1975 were, however, subsequently claimed to contain errors (Hemilä 1995; Hemilä 1996a). Furthermore, both Chalmers 1975 and Dykes 1975 placed considerable weight on the double-blind placebo-controlled trial carried out by Karlowski 1975a at the National Instiute of Health (NIH) , which concluded that a statistically significant benefit of vitamin C supplementation was caused by the placebo effect. It was subsequently argued that the placebo-explanation in the Karlowski 1975 paper was not consistent with their own data (Chalmers 1996; Hemilä 1996b; Hemilä 1996d).

Hemilä 1997b claimed that the highly cited reviews of Chalmers 1975 and Dykes 1975, and the Karlowski 1975a trial, quelled interest in the real, but modest effects of vitamin C on the common cold after the mid-seventies. Hemilä 1997c pooled the results of the six largest trials and found no effect on common cold incidence using 1 g/day or more of vitamin C (RR = 0.99; 95% CI 0.93 to 1.04). However, four trials with UK males found moderate reduction in common cold incidence by vitamin C (RR = 0.70; 95% CI 0.60 to 0.81) which was suggested to be caused by the particularly low dietary vitamin C intake in the UK rather than high supplement doses. Also, three trials with subjects under heavy acute physical stress had reported reduced incidence of colds with vitamin C (RR = 0.50; 95% CI 0.35 to 0.69) (Hemilä 1996b).

Although regular vitamin C supplementation at doses of 1 g/day or more has consistently decreased the duration or alleviated the symptoms of the common cold, there was substantial heterogeneity in the results (Hemilä 1994). In a further meta-analysis there was a trend for trials in children to show greater benefit than trials with adults, and another trend for trials where a dose was used of 2 g/day to show greater benefit than trials with 1 g/day of vitamin C (Hemilä 1999a).

In the first edition of this Cochrane review in 1998, an analysis was made of the 30 published trial comparisons that had been selected for attention by two previous systematic reviewers, Hemilä 1992 and Kleijnen 1989. That selection of trials was one of convenience and was justified by the fact that all had been carried out post-Pauling in an era of relatively sophisticated trial methodology, and mainly using doses of vitamin C at the level recommended by Pauling.

For this revised edition of the Cochrane review (2004) we have considered all known publications on the topic in the past 64 years including some trials that have been carried out since the earlier review. Twenty-five additional trial comparisons have been added to the review, including a number of trials which have evaluated the utility of vitamin C in the prevention of post-race colds among marathon runners and further explored the role of vitamin C as therapy for colds.

The terms 'common cold' and 'coryza' are used loosely both generally and in these trials. Most investigators have used self report by participants of a widely agreed constellation of symptoms and the self-assessed duration and severity of those symptoms, to evaluate the impact of vitamin C supplementation.

Three distinct evaluative approaches are discernible in the trials which have been conducted.
(1) Experimental prophylaxis trials in which volunteers were artificially exposed, in a laboratory setting, to known respiratory viruses, after preliminary dosage with vitamin C or placebo.
(2) Community prophylaxis trials in which volunteers took regular daily supplements of vitamin C or placebo over a study period ranging from weeks to months, in an effort to prevent the acquisition of colds and to ameliorate the effects of the colds that occurred. In some of these trials, medication was increased during the first few days of the colds that occurred.
(3) Community therapeutic trials which evaluated the therapeutic effects of vitamin C that was commenced only after naturally acquired cold symptoms had developed.

OBJECTIVES

The central question for the review is: "Does vitamin C in doses of 200 mg daily or more, reduce the incidence, duration or severity of the common cold when used either as continuous prophylaxis or at the onset of cold symptoms?"

CRITERIA FOR CONSIDERING STUDIES FOR THIS REVIEW

Types of studies

Placebo-controlled trials of vitamin C to prevent or treat the common cold using oral doses of vitamin C of 200 mg/day or more, and comparing outcomes with a suitable placebo preparation. The description of the study must enable it to be methodologically assessed using the Jadad quality score (Jadad 1996) and provide statistical data that could be entered into one or more of the five meta-analyses. These were the minimal criteria for inclusion of a trial in the review.

Types of participants

Trials of children and adults of either gender and any age were considered eligible.

Types of intervention

The only interventions considered were comparisons of orally administered vitamin C of at least 200 mg daily and a suitable placebo (which in a few instances included a low dose of vitamin C; Carr 1981a used 70 mg/day whereas a few others used 50 mg/day or less. This has been done by some investigators to ensure that participants were not vitamin C "deficient", recognizing that regular dietary intake of vitamin C is highly variable in some groups).

Types of outcome measures

"Incidence" of colds during prophylaxis was assessed as the proportion of participants experiencing one or more colds during the study period.
'"Duration" was the mean days of illness of cold episodes.
"Severity" of these episodes was assessed in two ways:days confined indoors or off work or school per episode and by symptom severity scores..
"Evidence of possible medication side effects" was availably from seven large prophylaxis studies where the number of subjects reported possible medication side effects in the active and control groups

SEARCH STRATEGY FOR IDENTIFICATION OF STUDIES

See: Cochrane Acute Respiratory Infections Group search strategy

The following electronic databases were searched for reports of trials: the Cochrane Central Register of Controlled Trials (CENTRAL) (*The Cochrane Library* Issue 2, 2004); MEDLINE (January 1966 to June 2004); and EMBASE (1990 to June Week 23 2004).
We ran the following search strings in combination with the search strategy developed by the Cochrane Collaboration for identifying randomised controlled trials (Dickersin 1994)

MEDLINE and CENTRAL were searched using the following search strategy:
1 exp Common Cold/
2 common cold$.mp.
3 exp RHINOVIRUS/
4 rhinovir$.mp.
5 or/1-4
6 exp Ascorbic Acid/
7 ascorbic acid.mp.
8 vitamin c.mp.
9 or/6-8
10 5 and 9

EMBASE search strategy:
1 exp Common Cold/
2 common cold$.mp.
3 exp Rhinovirus/
4 rhinovirus infection$.mp.
5 or/1-4
6 exp Ascorbic Acid/
7 vitamin c.mp.
8 or/6-7
9 5 and 8

We also screened the reference lists incorporated in a series of systematic reviews of the literature published by Briggs 1984 and Kleijnen 1989 (for the search strategies, see Kleijnan 1992) and the papers in those studies. One of the current reviewers (HH) has a fifteen year research involvement in this topic and has assembled a large personal reference list of papers published in the grey literature or listed in indexing services that preceded electronic searching. These were added to a primary database which was then systematically screened by two reviewers (RMD and RDS) who worked together to exclude duplicate entries, preliminary reports of data more fully reported elsewhere, commentaries, editorials and other papers which did not contain unique reports of controlled or randomized clinical comparisons.

These two reviewers then separately reviewed hard copies or electronic abstract data on each of 84 papers, applying the selection criteria outlined above. A final list of 62 candidate papers was selected which contained unique data from one or more trials of vitamin C and the common cold. One of these papers (Bibile 1966) remains uun assessed as we have been unable to retrieve a copy through library orders. Twenty-six of the 61 remaining papers failed to meet the selection criteria.

This left us with 36 papers, of which 12 contained reports of two or more (up to six) unique study comparisons and an entry for each comparison was made into the tables of included studies, using the letters a, b, c, d, e and f to identify different study comparisons within the one publication. This review includes data from 55 distinct trials , which is 25 more than in our earlier review. In four of the papers (Anderson 1974a; Anderson 1975a; Audera 2001a; Karlowski 1975a) more than one actively treated group was compared with the same placebo treated group. (To avoid the "unit of analysis problem" for which we were legitimately criticised in the earlier review, where multiple active arms were considered separately in the same meta-analysis, they were combined as one entry which appears in the figures, identified as the "highest" lettered trial that it contained.)

METHODS OF THE REVIEW

The circumstances and results of three small experimental prophylaxis trials were summarised in a separate table and were not included in the meta-analyses with the community based trials.

For the community trials, three outcomes were selected to compare vitamin C with placebo recipients, resulting in five meta-analyses; the number in parenthesis refers to the respective Comparison Figure in the Analyses:

(Comparison 1) "Incidence" - the proportion of participants who experienced one or more episodes of respiratory illness during prophylaxis;
(Comparison 2) and (Comparison 4) "Duration" - mean days of cold symptoms per illness episode (episodes occurring in trials of prophylaxis and therapy were analysed separately); and
(Comparison3) and (Comparison 5) "Severity" - mean severity score for the illness episode (also applied to both prophylaxis and therapy trials). The severity index was a continuous variable measured in two ways in different trials: a) the number of days that the patient was absent from work or school or confined to bed: and b) a symptom severity score derived from patient kept records

A meta-analysis was conducted using RevMan software for each of these five outcomes.

A pooled relative risk (RR) of the probability of experiencing one or more colds while taking vitamin C was computed for the incidence data. Because of the heterogeneity observed in this outcome across the trials, a random effects model in RevMan was applied to the pooled estimate. Heterogeneity was explored both qualitatively and using sensitivity analysis.

The pooled weighted mean difference (WMD) in illness duration was computed to derive an estimate of the percent of days of illness by which vitamin C reduced the average common cold. Because duration of cold episodes varied appreciably across trials, we standardised the mean values and standard deviations obtained in each trial group, against the mean of their placebo group. In this way, the placebo group gets value 100%, and the difference between vitamin C and placebo group is directly the effect of treatment in percentages. Because of the level of heterogeneity observed across trials we applied a random effects model to compute separate pooled estimates of the WMD for two sub-groups; adults and children.

Some trials presented the mean duration or severity of colds, but not the respective standard deviation (SD). In some trials the p-value for the difference of interest was reported and the SD was calculated from it. In case of the Anderson 1972 and Anderson 1974a and Anderson 1975a trials, Fieller's theorem was used to estimate the SD for individual common cold episodes from the SD values presented in papers that were based on per person experience. In the other trials with missing SD we estimated SD as identical with the mean of the treatment group. This is based on the analysis, that for trials reporting the SD, the ratio of SD to mean is on average 0.7 so that our ratio of 1.0 used in SD-estimation is somewhat conservative. The consequence of this is that we are putting slightly reduced weight in our estimates of effect on these trials with missing SD values, compared to the average.

The two different approaches to the assessment of severity were considered separately in the meta-analysis by treating the two sets of trials as separate subgroups. A standardized mean difference (SMD) was computed for each pool of results to enable us to derive a pooled estimate of the effect of vitamin C on cold severity across all trials on which severity data were available.

The SMD method leads to quantitative results that cannot be directly interpreted. Rather the primary statistical result of the SMD method is the p-value for the combined set.

Four factors were considered as possible explanations for the heterogeneity observed across the results of these trials. These were trial quality, vitamin C dosage, age of participants, and the particular life circumstances of the participants.

To explore the role of vitamin C dosage, each study comparison was categorised using the dose of vitamin C that active recipients were taking on the first day of development of respiratory illness:
(1) more than or equal to 200 mg and less than 1 g vitamin C per day;
(2) more than or equal to 1 g and less than 2 g vitamin C;
(3) from 2 g to 3 g of vitamin C;
(4) more than 3 g of vitamin C.

This variable was assigned to each meta-analytic study entry as a sorting variable in the RevMan software. It appears in the meta-analyses as the "user defined" variable. Where different study arms were combined in the analysis to compare with a single placebo group as part of our effort to avoid distortion of the pool estimate, the dose value assigned to the arm receiving the highest vitamin C dose was assigned to the combined group in the user defined variable. Doses for individual arms that are incorporated in a combined arm comparison are presented in the table of included studies

In the meta-analysis of duration while on prophylaxis, children and adults were considered as separate subgroups.

In analysing individual dichotomous data, we used Fisher's exact test. Two-tailed p-values are used in this review.

Assessing the role of study quality
To test the robustness of our conclusions regarding incidence and duration, we undertook a sensitivity analysis in which we first excluded from the analysis all of the studies in which allocation concealment was judged to be "inadequate" and then considered only those in which it was judged to be adequate (ii.e.leaving out of the analysis even those in which the judgment about allocation concealment was "uncertain" from the written evidence provided in the report of the study).

Unit of analysis issues
In the first edition of this review we were rightly criticized for a "unit of analysis" problem, as we compared several arms of a trial to a single placebo group, which meant that the same placebo groups was counted several times in pooling. In the current version we have combined the respective treatment arms to a single treatment group so that there is no inflation of participants in the placebo groups, as in the previous review. Miller and Carr studied twins, and this was pointed out by a comment on the previous version. Our SD values used in the calculations are based on SE and p-values, respectively, of paired tests, so the two trials are getting proper weight in pooling.

DESCRIPTION OF STUDIES

Fifty-five separate comparative studies reported in 36 publications met the selection criteria. Twelve of these publications presented the results of from two to six different study comparisons. Included in the selected papers are the four reports identified originally by

Pauling (Pauling 1971a; Pauling 1971b) to justify his proposals for mega dose prophylaxis and therapy (Cowan 1942; Franz 1956; Ritzel 1961; Wilson 1973a). We have used Wilson's more definitive 1973 reports of his boarding school studies rather than the preliminary communication of that group's first study which Pauling had available to him.

In Anderson 1974a , Anderson 1975a, Audera 2001a and Karlowski 1975a, more than one active arm is compared with a single placebo arm. This means that the total subjects presented in the summary analysis tables are less in the placebo groups than in the vitamin C groups.

The 55 included trials which have contributed data to this report fall into three distinct methodological groups:
(1) Three laboratory prophylaxis trials (Dick 1990; Schwartz 1973; Walker 1967) in which volunteers were intentionally exposed to known viruses after preliminary dosage with megadose vitamin C or placebo. Because they are small and qualitatively different from the community based studies they have not been included in the meta-analyses but are presented together in Table 1.
(2) Forty-one distinct community prophylaxis trials which evaluated the effects of daily supplementation with vitamin C on reducing the incidence and/or severity of naturally acquired colds.
(3) Eleven community therapeutic trials that evaluated the therapeutic effects of high dosage vitamin C after natural common cold symptoms had commenced.

Brief details of the circumstances, dosage, and quality assessment of all trials are available in the table of included studies.

METHODOLOGICAL QUALITY

Three indicators of study "quality" were collected on all trials.
(1) Allocation concealment in which a series of judgements based on explicit criteria are made relating to the question whether the assigned treatment was adequately concealed prior to allocation. Three categories were used: A: Adequate, B: Unclear, C: Inadequate.
(2) The Jadad score (Jadad 1996) which requires allocation of points out of five relating to the methodological statements in the text about 'blinding' of participants and investigators and the process of randomisation as well as the reporting of trial 'drop outs. The Jadad scores ranged from 0 to 5.
(3) Placebo distinguish-ability (PD) based on evidence presented in the publication as to the visual and taste characteristics and distinguish-ability between the test preparation of ascorbic acid and the placebo. The two categories were: I: placebo explicitly stated to be indistinguishable from vitamin C tablet, and ? : Uncertain, no explicit comments.

Study quality was not used as an exclusion criterion, but we only included trials in the meta-analyses which were sufficiently well documented to enable us to assign values for each of the three measures of study quality. Allocation concealment was used to sort the meta-analyses, when exploring possible reasons for study heterogeneity and sensitivity analysis was carried out to test the robustness of the findings of the review when the meta-analyses were confined to studies in which allocation concealment was judged to be adequate.

Allocation concealment, Jadad scores and Placebo Distinguishability assessments are presented in the tables of included trials.

RESULTS

a) Prophylaxis trials in artificially infected volunteers
Three prophylaxis trials were volunteer transmission studies which are summarised in Table 01. Walker 1967 and Schwartz 1973 instilled virus into the noses of volunteers who had been pre-treated with vitamin C or placebo, whereas Dick 1990 used a more natural mechanism for

transmission of a known rhinovirus. Their volunteers were housed for a week and worked closely with volunteers who had been previously infected by nasal instillation of rhinovirus. In these Dick studies, less of the vitamin C treated volunteers became infected and the cumulative symptom severity score and mucus weights were significantly less ($p = 0.03$), although the virus shedding was similar in both treatment groups. Schwartz found reduced common cold severity in vitamin C group ($p < 0.02$ at day 4), but no effect on symptom duration, whereas Walker failed to report any benefit to those who took vitamin C.

Table 1 Trials using artificial infection of common cold

Study Characteristic	Walker 1967	Schwartz 1973	Dick 1990
Number of participants	91 healthy volunteers; 47 vit C and 44 placebo	21 healthy male volunteers 11 vit C and 10 placebo	Three separate experiments each involving 16 healthy volunteers (8 vit C 8 placebo) housed closely for one week with 8 volunteers actively infected with rhinovirus. Altogether 24 vitamin C and 24 placebo recipients.
Viruses used	Rhinovirus (3 strains) 29/26<br>Influenza B 8/8<br>B814 virus 10/10	Rhinovirus 44	Rhinovirus 16
Transmission method	Nasal instillation	Nasal instillation	Close contact with infected volunteers over a period of a week
Vitamin C Intervention	1 g vit C or placebo three days before and six days after inoculation	3 g vit C or placebo daily for two weeks before and one week after instillation	2 g vit C or placebo daily for 3.5 weeks before exposure to infected volunteers
Incidence outcome	18 colds developed in each group	10 colds developed in both groups	19 of 24 vitamin C and 22 of 24 placebo recipients developed symptoms of colds
Duration outcome	Mean of five days duration for each group	Both groups fully resolved by day six or seven	Not provided
Severity outcome	Mean severity score 8 for vitamin C and 7 for placebo	Severity peaked earlier for the vitamin C group and resolution significantly more advanced by day four (P < 0.02), but overall mean severity scores not significantly different in the two groups.	Mean cumulative severity score and mucus weights significantly reduced in the vitamin C recipients (P = 0.03)
Comment	Not double-blind	Double-blind. Nasal virus shedding similar in the two groups	Double-blind. Virus shedding similar in the two groups.

b) Community prophylaxis trials: "incidence" of colds

Comparison 01 presents the meta-analysis of the relative risk of one or more colds developing while on prophylaxis. The entry in the meta-analysis for Anderson 1974a represents four separate study arms (Anderson 1974a; Anderson 1974b; Anderson 1974c; Anderson 1974d) in which different vitamin C dosages ranging from 250 mg daily to 2 g/day were compared with one placebo group. Thus the 29 entries in the figure represent 32 trial comparisons.

Review: Vitamin C for preventing and treating the common cold
Comparison: 01 Development of colds while on prophylaxis
Outcome: 01 Proportions developing one or more cold episodes during prophylaxis

Study or sub-category	Treatment n/N	Control n/N	Weight %	RR (random) 95% CI	Year	Quality	Order
01 All eligible trials with exception of subgroup removed below							
Peters 1996b	5/41	11/45	0.20	0.50 [0.19, 1.31]	1996	B	1
Moolla 1996b	5/11	12/19	0.34	0.72 [0.35, 1.50]	1996	B	1
Charleston 1972	31/47	37/43	2.75	0.77 [0.60, 0.97]	1972	C	2
Coulehan 1974a	19/190	23/192	0.55	0.83 [0.47, 1.48]	1974	C	2
Anderson 1972	302/407	335/411	10.77	0.91 [0.85, 0.98]	1972	A	4
Coulehan 1974b	16/131	17/128	0.45	0.92 [0.49, 1.74]	1974	C	2
Dahlberg 1944	131/1259	142/1266	3.03	0.93 [0.74, 1.16]	1944	B	1
Bancalari 1984	21/32	21/30	1.46	0.94 [0.67, 1.32]	1984	B	3
Franz 1956	14/44	15/45	0.51	0.95 [0.52, 1.74]	1956	A	1
Anderson 1974a	922/1191	233/285	11.75	0.95 [0.89, 1.01]	1974	A	4
Cowan 1942	184/208	142/155	11.24	0.97 [0.90, 1.03]	1942	B	1
Ludvigsson 1977b	230/304	240/311	9.52	0.98 [0.90, 1.07]	1977	B	2
Pitt 1979	298/331	309/343	12.89	1.00 [0.95, 1.05]	1979	A	3
Coulehan 1976	98/428	98/428	2.61	1.00 [0.78, 1.28]	1976	A	2
Clegg 1975	48/67	50/70	3.33	1.00 [0.81, 1.24]	1975	B	2
Elwood 1976	296/339	298/349	12.06	1.02 [0.96, 1.09]	1976	B	2
Van Straten 2002	35/84	34/84	1.32	1.03 [0.72, 1.48]	2002	A	2
Carson 1975	85/121	84/123	4.72	1.03 [0.87, 1.22]	1975	C	2
Briggs 1984	125/265	121/263	4.16	1.03 [0.85, 1.23]	1984	A	2
Ludvigsson 1977a	49/80	44/78	2.35	1.09 [0.84, 1.41]	1977	B	2
Liljefors 1972	10/33	9/33	0.32	1.11 [0.52, 2.38]	1972	A	3
Peters 1993b	18/34	18/39	0.83	1.15 [0.72, 1.82]	1993	C	1
Himmelstein 1998b	10/23	8/25	0.34	1.36 [0.65, 2.84]	1998	A	2
Subtotal (95% CI)	5670	4765	97.50	0.98 [0.95, 1.00]			
Total events: 2952 (Treatment), 2301 (Control)							
Test for heterogeneity: Chi² = 17.73, df = 22 (P = 0.72), I² = 0%							
Test for overall effect: Z = 1.90 (P = 0.06)							
02 Short term exposure to cold and/or severe physical stress							
Peters 1996a	7/44	19/47	0.32	0.39 [0.18, 0.84]	1996	B	1
Sabiston 1974	6/56	14/56	0.24	0.43 [0.18, 1.04]	1974	C	2
Moolla 1996a	4/13	13/19	0.25	0.45 [0.19, 1.07]	1996	B	1
Peters 1993a	14/43	28/41	0.79	0.48 [0.30, 0.77]	1993	C	1
Ritzel 1961	17/139	31/140	0.62	0.55 [0.32, 0.95]	1961	A	2
Himmelstein 1998a	10/30	6/14	0.30	0.78 [0.35, 1.71]	1998	A	2
Subtotal (95% CI)	325	317	2.50	0.50 [0.38, 0.66]			
Total events: 58 (Treatment), 111 (Control)							
Test for heterogeneity: Chi² = 1.93, df = 5 (P = 0.86), I² = 0%							
Test for overall effect: Z = 4.98 (P < 0.00001)							
Total (95% CI)	5995	5082	100.00	0.96 [0.92, 1.00]			
Total events: 3010 (Treatment), 2412 (Control)							
Test for heterogeneity: Chi² = 45.35, df = 28 (P = 0.02), I² = 38.3%							
Test for overall effect: Z = 2.00 (P = 0.05)							

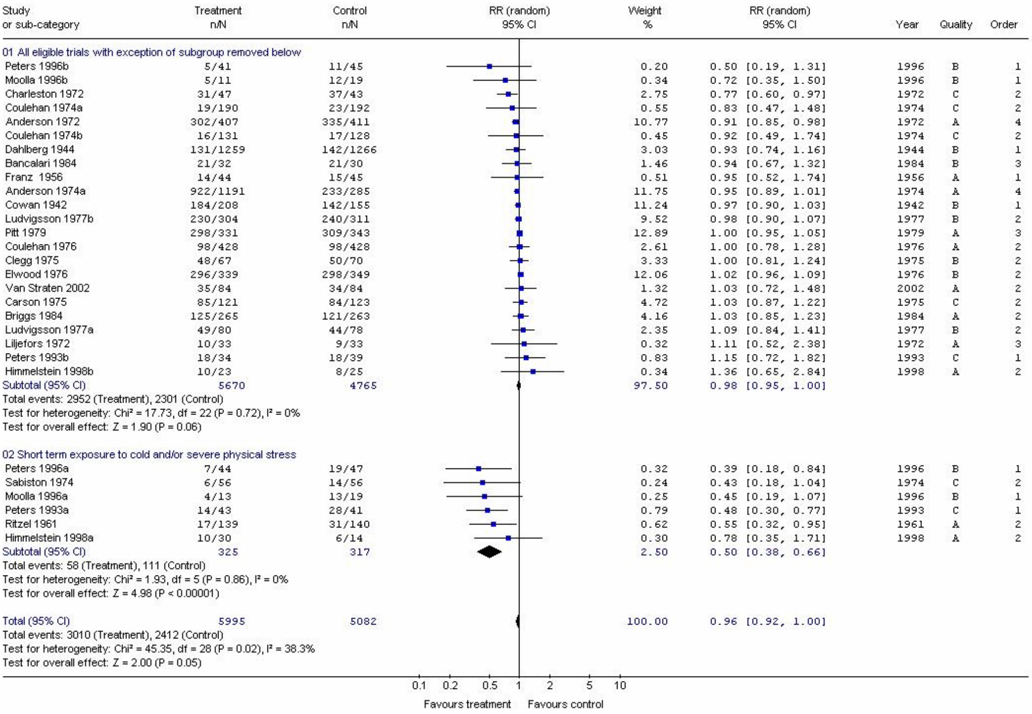

The studies summarised here represent 11,077 participants, of whom 5,995 used vitamin C for periods ranged from two weeks to six months and the RR of developing a cold while taking vitamin C prophylaxis in individual trials ranged from 0.39 to 1.36. The pooled RR for all trials using a random effects model (because of the significant heterogeneity of the results), was 0.96 (95% CI 0.92 to 1.00).

Heterogeneity of results

Among all the 29 entries included in Figure 1 there is substantial heterogeneity, as indicated by the chi square test (p = 0.02) and the high I squared value .

Five of the 32 trials recorded statistically significant ($p < 0.05$) protection favouring the vitamin C group: Peters 1996a (RR = 0.39), Peters 1993a (RR = 0.50), Ritzel 1961 (RR = 0.55), Charleston 1972 (RR = 0.77), and Anderson 1972 (RR = 0.91). Five other trials recorded a non-significant RR = 0.80 (Himmelstein 1998a; Moolla 1996a; Moolla 1996b; Peters 1996b; Sabiston 1974).

None of the 32 trials significantly favoured the placebo but one reported a RR = 1.2.

Of the nine relatively small trials with RR < 0.8, 4 were in marathon runners (Himmelstein 1998a; Moolla 1996a; Peters 1993a; Peters 1996a), two others were in controls for marathon runners, (Moolla 1996b; Peters 1996b), one was in students in a skiing school in the Swiss Alps (Ritzel 1961), one was in Canadian army troops on subarctic operations (Sabiston 1974), and one in staff and students at Glasgow University, UK (Charleston 1972). A subgroup analysis is shown in Figure 1 in which the six studies which involved marathon runners, skiers, and Canadian soldiers in a subarctic exercise were moved to a separate subgroup in the meta-analysis. This resulted in two distinct groups of trials which were significantly different from each other in their pooled estimates of effect. Furthermore, the two subgroups were not heterogeneous within the two pools, as indicated by the high p-values in chi-square test, and the zero values for the I square value.

All of these six physical and/or cold stress studies were randomised controlled trials. For three of them, the dose of vitamin C used as prophylaxis was less than 1 g daily so that the effect in this subgroup is not explained by the highest doses.

To test the effect of study quality on the findings, we undertook sensitivity analysis in which we first removed from the meta-analyses the seven study entries in which allocation concealment was judged inadequate. Total pooled RR was 0.97 (95% CI 0.94 to 1.01), with the pooled RR value for the physical and/or cold stress studies at 0.55 (95% CI 0.37 to 0.76). We then further removed the 17 study entries in which the judgment on allocation concealment was "uncertain" from the available evidence. This left entries with a total pooled RR 0.97 (95% CI 0.94 to 1.00) and the two remaining studies in the physical and/or cold stress pool with a RR 0.62 (95% CI 0.39 to 0.96). Thus, the effect of study quality as assessed by allocation concealment in this meta-analysis did not appreciably change either the quantitative estimates of the pooled results, or the qualitative conclusions.

c) Community prophylaxis trials: duration of colds

The meta-analysis in **Comparison 02** on duration of colds which developed while subjects were taking prophylaxis, contains two subgroups, adults and children. For adults there were 18 entries representing 22 trial comparisons (four separate trial arms in one entry for Anderson 1974a and two for Karlowski 1975a) and 7,242 episodes of illness, and for children there were 12 trial comparisons including 2,434 episodes of illness. The division into subgroups of child and adult trials was carried out for two reasons: A) children have substantially higher incidence of colds reflecting differences in immune system maturity, and B) children are on average smaller so that a fixed dose corresponds to a greater dose per weight.

Quite consistent benefit was seen in duration of colds, but the effect was greater in the children. For children, the pooled effect was 13.6% (95% CI 5.5% to 21.4%) reduction in common cold duration, and for adults, the pooled effect was 8.0% (95% CI 3.0% to 13.1%) reduction in duration. Within neither group was the Chi square test for trial heterogeneity statistically significant.

All but four of the 30 comparisons (Carr 1981a; Himmelstein 1998a; Peters 1993a; Wilson 1973b) recorded a point estimate favouring the vitamin C group. Wilson 1973b used only 200 mg/day vitamin C, which is the smallest dose in the table. Carr 1981a examined twins living together, whereas the Carr 1981b trial examined twins living apart; it is possible that the substantially divergent result in these groups is related to the living conditions, e.g. those living together might conceivably have exchanged or confused their tablets. In discord with all the other trials, Himmelstein 1998a recorded in their marathon runners a statistically significant increase in common cold duration by vitamin C (though incidence was decreased in the vitamin C takers.) There was an extreme and divergent drop-out rate in the Himmelstein 1998a trial. They started with 52 marathon runners in two groups, but 42% (22 of 52) of the vitamin C group, and 75% (38 of 52) of the placebo group dropped out during the trial (p = 0.003). The apparent increase in common cold duration might be related to biases caused by the high and significantly divergent drop-out rate. In a sensitivity analysis, we excluded this divergent Himmelstein 1998a trial from the adult subgroup, and there was a substantial reduction in the heterogeneity (p = 0.55 in the chi square test; and I2 = 0%), and the test for overall effect in this adult subgroup became even more significant (p = 0.0002). In five of the 30 trials (Carr 1981b; Charleston 1972; Ludvigsson 1977a; Peters 1993b; Ritzel 1961), the episode duration difference was statistically significant within the trials themselves.

The great majority of the trials in Figure 2 used 1 g/day of vitamin C and therefore a systematic examination of possible dose-dependency across the trials was not feasible. We used sensitivity analysis to test the possible role of low vitamin C doses in affecting the estimate of effect in the child subgroup. When we removed the trials using less than 1 g/day of vitamin C (Miller 1977b; Miller 1977c; Wilson 1973a; Wilson 1973b), the pooled estimate of benefit was increased to 18.5% (95% CI 7.3% to 29.7%).

To test the effect of study quality on the findings, we undertook sensitivity analysis in which we first removed from the meta-analyses the studies in which allocation concealment was judged "inadequate". Total pooled benefit for adults was 7% (95% CI 1% to 13%), and the pooled benefit for children was 15% (95% CI 4% to 25%). When we further removed the studies in which the judgment was "uncertain" from the available set of trials, the benefit indicated by the remaining studies for adults was 8% (95% CI 1.5% to 17%) and for children

15% (95% CI 3% to 34%). Thus, the effect of study quality as assessed by allocation concealment in this meta-analysis did not appreciably change either the quantitative estimates or the qualitative conclusions.

In summary, this meta-analysis of duration of colds experienced while subjects were taking prophylaxis demonstrated a modest but consistent and statistically significant benefit to the vitamin C supplemented participants which was greater in children than adults.

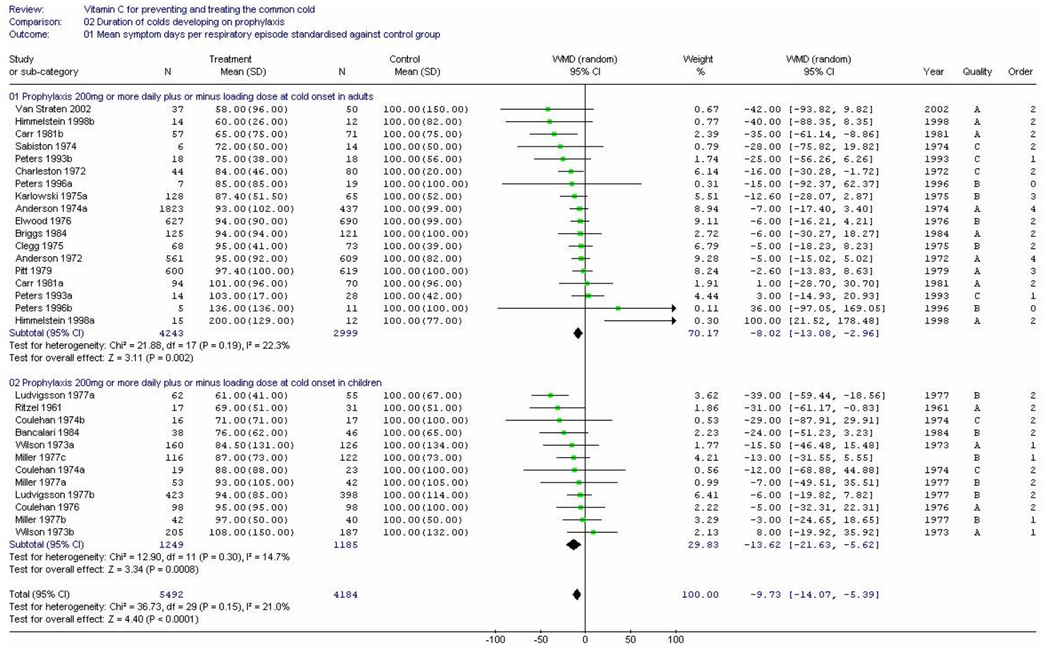

d) Community prophylaxis trials; severity of colds

Two types of measures of the severity of illness were available. Seven entries in **Comparison 03** present the results of 10 vitamin C study arms in which severity was measured by 'days confined to home' or 'days off work or school' (subgroup 1). This included 5,066 respiratory episodes The large scale trial by Anderson 1972 reported a statistically significant protection for vitamin C contributing to a modest, but significant reduction for the pool as a whole, which included both adults and children. This subgroup exhibited highly significant heterogeneity across the subgroup as measured by the chi square and I square tests.

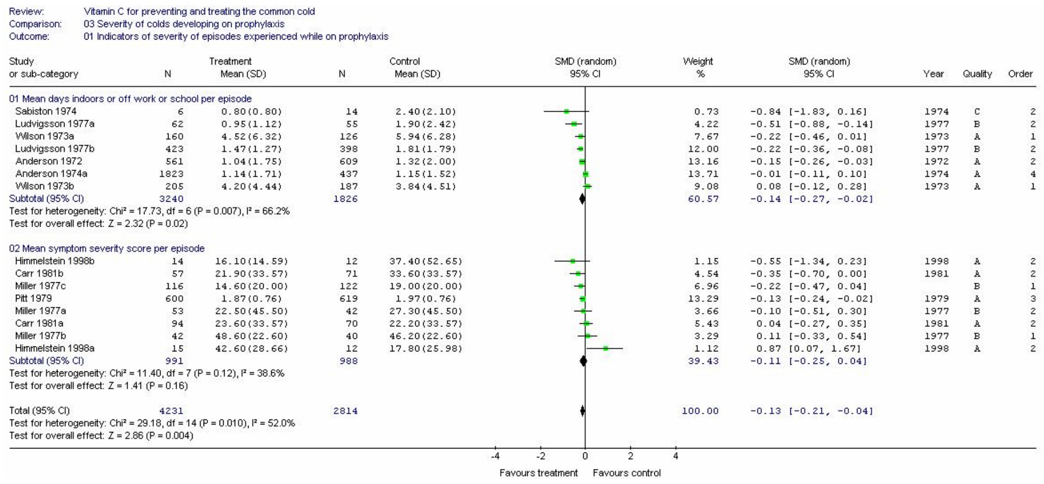

Subgroup 2 in Figure 3 presents the results of symptom severity scores in eight trials. The large scale trial by Pitt 1979 found a statistically significant, but small 5% reduction in severity

score. Here too, the subgroup exhibited highly significant heterogeneity across the subgroup as measured by the chi square and I square tests. Himmelstein 1998a found substantially greater severity in vitamin C administered marathon runners, but as noted above, this trial had particularly high and divergent drop-out rate, and the study groups may be biased. In a sensitivity analysis, excluding the Himmelstein 1998a trial substantially reduced the heterogeneity among the remaining seven trials (p = 0.42 in chi square test, and I2 = 0.9%), and the overall effect significantly favoured vitamin C in this subgroup (p = 0.0009).

The measures of 'severity' that have been used in the trials are highly variable and we used the standardised mean difference which normalizes the results to standard deviations. Therefore the pooled results of Figure 3 are not practically useful, rather, the significance level is of main importance in this case; p = 0.03 for the studies that assessed days confined to home or off work or school, and p = 0.09 for studies which used severity scores, and p = 0.003 when the two pools using different measures of severity were combined.

Sensitivity analysis using allocation concealment as the excluding variable failed to change appreciably the standardised mean difference that was estimated from the whole pool.

In summary, there was inconsistent evidence of the benefit of vitamin C on the severity of illness episodes that were experienced during prophylaxis. Such benefit with respect to days confined to home or off work or off school as was observed was statistically significant, but relatively slight in absolute terms which can be seen by viewing the original mean values in the figure.

e) Community therapeutic studies: duration of colds in which vitamin c or placebo were commenced after cold symptoms began.

The meta-analysis presented in **Comparison 004** contains 76 entries that incorporate data from 11 different trial arms involving 3,294 cold episodes where participants initiated supplementation at the onset of cold symptoms. Audera 2001a contains three different vitamin C dosage arms, while Anderson 1974e and Anderson 1975a each contain two different vitamin C dosage arms. These are detailed in the table of included studies.

The pooled result for these therapeutic trials, unlike that seen in the prophylaxis trials, did not exhibit a consistent difference of vitamin C from placebo in the variety of therapeutic protocols that were used. The large trial by Anderson 1974e found statistically significant but modest benefit on severity but this was counterbalanced by the negative results in other trials.

The statistically significant Anderson 1974e entry combined two different dosage arms. Anderson 1974e administered 4 g/day, and Anderson 1974f administered 8 g/day at first day of illness only. The mean duration of illness episodes for those in the 4 g/day arm was 3.17 days, while that for 8 g/day arm was 2.86 days compared with the duration in the placebo group #4 with 3.52 days. This 1974 trial was bbedeviledhowever by the fact that the investigators originally intended to compare results with two separate placebo groups. One of the placebo groups (#6) had substantial baseline differences when compared with the six vitamin C groups. The comparisons presented here are with the placebo group #4 that was much closer to the vitamin C groups with respect to baseline data. Were the comparison to be

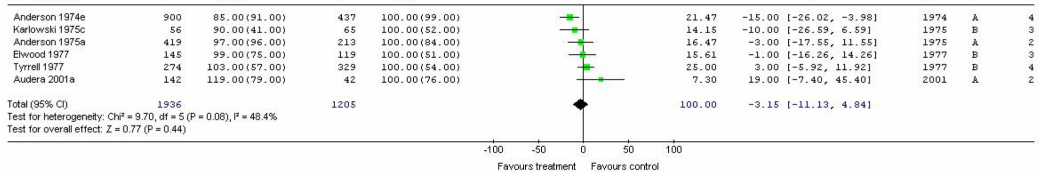

made either with the placebo group #6 or a combination of the two placebo groups as the investigators originally intended, the benefits would have been minimised as the mean episode duration for the placebo group #4 was 3.52, and for placebo group #6 was 2.83, and

for the combination of two placebo groups was 3.18 days. Nevertheless, independently of the placebo group problem, the proportion of "short colds," that lasted for only 1 day was larger in the 8 g/day group (46%; 222/483) compared with the 4 g/day group (39%; 164/417) (p = 0.04), consistent with the possibility of therapeutic benefit at the higher dosage.

Tyrrell 1977, Elwood 1977 and Audera 2001 failed to show an effect on duration. Tyrrell evaluated separately males and females using a dosage of 4 g/day for the first 2.5 days of illness (total 10 grams), Elwood evaluated separately males and females using a dosage of 3 g/day for the first 3.3 days of illness (total 10 grams), and Audera evaluated 3 g/day over the first 3 days (total 9 grams).

Sensitivity analysis in which allocation concealment was used as the excluding variable, once again failed to change the conclusions of this meta-analysis.

In summary, the data from the therapeutic trials do not provide convincing evidence of reduced duration with the protocols that have been tested and the apparent benefits from use of an 8 g single dose may be regarded as "equivocal".

f) Community therapeutic studies; severity of cold episodes when vitamin C or placebo were commenced after cold symptoms began

Comparison 05 has four entries which represent 8 trial arms that included 2,753 separate respiratory episodes for which cold severity was assessed. (Anderson 1974a and Anderson 1975a contain two vitamin C arms and Audera 2001 contains three different vitamin C arms). As with the prophylaxis studies, we have separated the measures of severity into two different subgroups (days confined to home, off work or school and symptom severity scores) and analysed the subgroups separately and together.

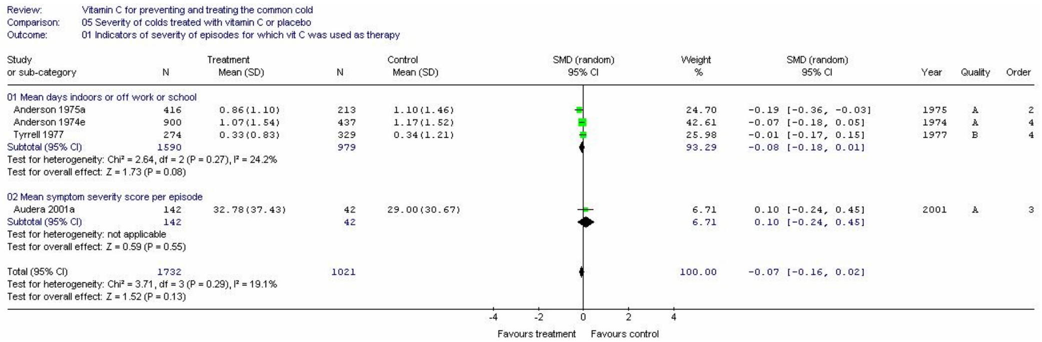

In the first subgroup, the only comparison which revealed marginally significant benefit to those taking vitamin C was that for Anderson 1975a. In both of the vitamin C arms, participants took 1.5 g/day for the first day of the common cold and 1 g/day for the following four days (total 5.5 grams). Anderson 1974e and Tyrrell 1977 found no meaningful difference between vitamin C and placebo. In the second group, the Audera 2001 trial similarly found no meaningful difference between vitamin C and placebo.

Once again, the conclusions did not change when carrying out sensitivity analysis based on allocation concealment.

In summary, therapeutic vitamin C supplementation has shown no convincing effect on common cold severity with the protocols that have been used.

g) Adverse effects from high dose vitamin C intake

Seven investigators of large prophylaxis trials recorded data on symptoms which participants attributed to the medication they were using.

Trials involving altogether 2,490 recipients who had used more than 1 g daily of vitamin C during prophylaxis compared with 2,066 who took a placebo recorded these data. Altogether

5.8% of the vitamin C recipients reported symptoms which they attributed to the medication compared with 6.0% of those who were taking placebo (data not shown).

No serious symptoms were reported.

DISCUSSION

The term 'the common cold' does not denote a precisely defined disease, yet the characteristics of this illness are familiar to most lay-people. Medically, it is a complex condition caused by a broad range of viruses that are transmitted in varying ways. There is no unanimously accepted definition for the condition that can be used for the practical definition of outcome in community based controlled trials. Instead, various authors have used different operational definitions such as a minimum set of symptoms. This variation in outcome definition could be contributing to heterogeneity in results, but we have not been able to explore this possibility.

Although the importance of the placebo-effect has been challenged (Hrobartsson 2001) we considered that with the expected small effects of vitamin C, and the greatly subjective outcome definitions, only placebo-controlled trials could yield information of adequate rigour to meet our study objectives. Most of the trials analysed in this review were reported to be double-blind, but that was not used as a selection criterion. Also we did not restrict the review to trials using random allocation and there are some trials included which had alternative allocation. Sensitivity analysis indicated that a restriction to trials for which requirements of allocation concealment were known to be met, did not alter the principal conclusions from our overview.

Despite the variation in methodology and the substantial heterogeneity in results from this large number of trial results carried out over a sixty year period, a rather coherent story has emerged from the review.

Effect on common cold incidence
Consistent with earlier reviews (Hemilä 1997c, First Edition of Cochrane Review 1998) we found no convincing reduction in common cold incidence in the prophylaxis trials when the subgroup of marathon runners and skiers and soldiers on su-arctic operations were excluded from the trial pool (RR = 0.98; 95% CI: 0.95 to 1.00).

A previous meta-analysis identified three trials with participants under severe acute physical stress which had found significant benefit from vitamin C supplementation (Hemilä 1996b), The more recent trials by Peters 1996a, Moolla 1996a and Himmelstein 1998a have reinforced and extended those observations. The small study reported by Sabiston 1974 which involved troops engaged in brief exercises in subarctic conditions, shares with this group of trials a low relative risk and a benefit that borders on significance. It is noteworthy that all of the studies in this group, involved brief exposure to high physical and/or cold stress and that they were not uniformly using high doses of vitamin C.

One of us (Hemilä 1997c) has also previously drawn attention to the possibility that some of the earlier benefits observed in low dose or controlled trials without a placebo, which were ruled ineligible for this review (Baird 1979; Glazebrook 1942), might be a consequence of suboptimal dietary intakes in British males. This might also explain the significant reported benefits in the Charleston 1972 study though participants in that study were single-blind and not randomized. Few of the recent trials have estimated the dietary intakes of vitamin C, but we cannot ignore the fact that vitamin C is an essential nutrient and all participants in the trials had regular intakes of this substance at some level, some of them with lower levels than others.

The large, well conducted trial by Anderson 1972 reported a statistically significant but quite small reduction in common cold incidence. This trial was conducted during winter in Toronto, Canada, and participants were selected on the basis of having had problems with colds

during previous winters. The relative risk observed in that trial was 0.91, the risk difference was 0.07, and thereby the number needed to treat (NNT) estimated from the study was 14. A cold Canadian winter might be a part explanation for the benefit in this trial if it is true that cold as well as physical stress makes a prophylactic benefit for vitamin C more likely. Furthermore, as regards the possible interaction between supplementation and dietary vitamin C levels, this Anderson 1972 trial is interesting as the investigators found 48% reduction by vitamin C in "total days indoors" among participants who consumed <3 oz of fruit juices, whereas vitamin C reduced total days indoors by only 22% among those who consumed more juices. Similar modifying effect by fruit juices was found in the therapeutic trial by Anderson 1975.

Effect on common cold duration and severity: prophylaxis trials

Both in adults and in children, regular vitamin C supplementation resulted in a statistically highly significant reduction in the duration of respiratory episodes that occurred during the prophylactic supplementation period. For children, the pooled estimate was 13.6%, and for adults it was 8.0%.

Although these findings point to a definite physiological effect by prophylactic vitamin C on common cold duration, the practical significance of these findings is less convincing. It would not seem reasonable to ingest vitamin C regularly in the mega-dose range throughout the year if the only anticipated benefit is to rather slightly shorten the duration of colds which occur for adults, two or three times per year. Our pooled estimate suggests that long term supplementation might result in an upper estimate average reduction of annual common cold morbidity from about 12 days (based on Douglas 1979; unpublished Australian data) to about 11 days per year for adults. For children under 12, who experience colds more frequently (on average for this age, the upper estimate could be as high as 28 days of cold morbidity annually, our pooled estimate of benefit suggests that long term prophylaxis might be associated with an average reduction in four symptom days from about 28 days to 24 days per year per child. Such a benefit is not trivial, but is it worth the cost of long term prophylaxis, and could an equivalent benefit perhaps be achieved in children through therapy alone?

In view of the consistent effect of vitamin C on the duration of colds, an evident question is whether there might be dose dependency, as suggested in a previous overview (Hemilä 1999a) that might translate to a benefit when vitamin C is used therapeutically. However, across the available pool of trials, duration would appear to be more determined by the nature of the participants than by dose. There are few trials that have used more than 1 g/day in the child and adult groups separately. Nevertheless, Karlowski 1975 and Coulehan 1974 used two different doses within the same trials, i.e. with the same outcome definitions. Karlowski's paper shows that for adults 6 g/day was associated with a double benefit compared with 3 g/day, and Coulehan 1974 found that for schoolchildren 2 g/day caused about twice the benefit of 1 g/day (Hemilä 1996b; Hemilä 1999a). Although these findings do not establish dose dependency, they support the case for examination of higher doses.

Regular vitamin C prophylaxis also led to some decrease in severity when measured as days indoors or days off work or school, but the effect was not unambiguous on severity score scales (Figure 3). These measures of severity are substantially more heterogeneous than the measures of symptom duration and the number of trials reporting data pertinent to 'severity' is small.

On the issue of the severity of colds, the Pitt 1979 paper is of further interest. This was a randomized placebo-controlled double-blind trial with 674 marine recruits during an eight week period using 2 g/day of vitamin C. There was no difference in common cold incidence and only a 2% reduction in duration of colds and 5% reduction in severity ($p = 0.023$). However, eight of the recruits developed pneumonia as a sequel to their colds and only one of these was in the vitamin C group ($p = 0.077$). Thus, in addition to the common cold, vitamin C might also affect other respiratory infections either independently of colds, or as complications of colds (Hemilä 1999b).

Effect on common cold duration and severity: therapeutic trials

Because the prophylaxis trials have relatively consistently shown that vitamin C affects duration and, to some extent, the severity of the common cold, without changing their

incidence in the normal population, it might seem rational to administer vitamin C therapeutically, starting immediately after the first symptoms. But the therapeutic trials that have evaluated this have been negative (Figures 4 and 5). The pooled estimates for duration and severity do not find any difference between vitamin C and placebo.

Technically the therapeutic trials are in some ways more complicated than regular supplementation trials. If the timing of initiation or the duration of supplementation affect the benefit, false negative findings might result.

Cowan 1950 used a therapeutic dose of 6g in the first two days of illness with no effect on duration. Elwood 1977, Tyrrell 1977, and Audera 2001 used a three day supplementation, and these three trials found no effect by vitamin C. A five-day therapeutic trial by Anderson (1975) found a reduction in 'days spent indoors per subject' because of illness by 25% ($p = 0.05$) in the vitamin C group (1-1.5 g/day). Also, using a five-day therapeutic supplementation of 3 g/day in a 2x2 factorial design trial, Karlowski (1975) reported that colds were 0.73 days shorter ($p = 0.10$; see Hemilä 1996b). These tfindingsare consistent with the possibility that three days might be too short a time for vitamin C to produce unambiguous benefits, and it seems that possible future therapeutic trials should use longer than three day supplementation.

Also, the possibly larger effect observed by 8 g compared with 4 g as a single dose in the Anderson 1974 trial would seem to suggest that future therapeutic trials with adults should use doses larger than 4 g per day.

Furthermore, none of the therapeutic trials have examined the effect of vitamin C on children, although the effect of prophylaxis on duration has been substantially greater in children compared with adults, and children have substantially higher incidence of acute respiratory tract infections.

Experimental prophylaxis trials

The summary evidence from the three experimental studies, which differed in their method of exposing volunteers to the infecting virus is instructive. The studies by Dick and his colleagues which have only been reported in conference proceedings, paid careful attention to the severity of the colds experienced by those who acquired them from fellow volunteers who had been inoculated with a known rhinovirus. They also found that in these more natural circumstances of acquiring the virus, less, but not significantly less, volunteers on vitamin C developed cold symptoms but demonstrated similar viral shedding in the vitamin C group. The tantalisingly fragmentary descriptions of the Dick studies show clearly a biological effect of high dose vitamin C on the nature and course of symptoms encountered. The findings appear to confirm the view from the community prophylaxis studies that the protective benefit from vitamin C comes into play after the virus has become established.

Pauling's contribution

Among the four trials included in Pauling's (Pauling 1971a) meta-analysis, the largest dose, 1 g/day, was used by Ritzel (Ritzel 1961). Pauling based his optimistic quantitative expectations on this rather small and brief trial. Ritzel found significant reduction in the incidence (-45%) and duration (-31%) of colds, and Pauling derived a combination of the duration and incidence, which he labelled 'integrated morbidity' referring to the total sickness days per person during the trial.

This was reduced by 61% in the Ritzel trial. Pauling (Pauling 1971a) used these Ritzel findings to extrapolate the effects of vitamin C to a broader community. The present analysis suggests that 'integrated morbidity' is not a good outcome measure, since the effects on incidence and duration/severity seem to have quite different patterns though in the case of the Ritzel study they moved together.

Further, Ritzel carried out his trial with schoolchildren in a skiing school in the Swiss Alps, and such children are not a representative selection of the general population, even though technically the trial was randomized, double-blind and placebo-controlled. In our analysis,

Ritzel's trial is included in the group of trials exposed to short lived severe acute physical stress and/or cold environment which highlights the special character of this trial.

Pauling's vigorous advocacy was undoubtedly the stimulus for a wave of good trials, which now enable us to better understand the rather confusing role that this substance plays in defence against the common cold. Significant uncertainties still persist, which further research could help to elucidate.

REVIEWERS' CONCLUSIONS

Implications for practice

The lack of effect of prophylactic vitamin C supplementation on the incidence of common cold in normal populations throws doubt on the utility of this wide practice. In special circumstances, where people are engaged in extreme physical exertion and/or exposed to significant cold stress the current evidence indicates that vitamin C supplementation may have a considerable beneficial effect, but caution should be exercised in generalizing this finding that is mainly based on marathon runners.

The prophylaxis trials found 8% reduction in common cold duration in adults, and 13.6% reduction in children, but the practical relevance of these findings are open, since the therapeutic trials carried out so far have not found benefits and this level of benefit probably does not justify long term prophylaxis in its own right.

In summary, on the basis of our analysis, there seems no justification for routine mega-dose vitamin C supplementation in the normal population. Prophylaxis may be justified in those exposed to severe physical exercise and/or cold. So far, therapeutic supplementation has not been shown to be beneficial.

Implications for research

With the findings from our analyses, it does not seem worth while to carry out further regular prophylaxis trials in the normal population. However there will be value in better understanding the role of vitamin C in those exposed to heavy exertion and cold stress. The findings in marathon runners, skiers and soldiers operating in sub-arctic conditions warrant further research.

None of the therapeutic trials carried out so far has examined the effect of vitamin C on children, even though the prophylaxis trials have found substantially greater effect on duration in children. In view of the greater incidence of respiratory infections in children such therapeutic trials are warranted, especially where there is known to be sub-optimal dietary intake of vitamin C.

The findings in the Anderson 1974 studies on the therapeutic use of very high doses of 4 g and 8 g on the day of onset of respiratory symptoms are tantalising and deserve further assessment in the light of the uncertainties raised by the problems with the placebo groups in that important study.

ACKNOWLEDGEMENTS

Professor Charles McGilchrist and Dr Keith Dear provided valuable statistical advice, and Ms Robyn Savory and Ms Leonie Hoorweg undertook a range of assistant duties in the first publication of this review. Mr Bob Galloway provided translations for a number of papers for us. English translations of the Ritzel (1961), Bessell-Lorck (1959), and Bancalari (1984) papers was kindly arranged by Eva Wintergerst from Roche Consumer Health LTD, Kaiseraugst, Switzerland.

POTENTIAL CONFLICT OF INTEREST

Professor Bob Douglas was coordinating investigator on the Audera 2001 study. None of the other authors has any conflict of interest to declare in this review.

TABLES

Characteristics of included studies

**Study**	**Anderson 1972**
Methods	Double blind randomised prophylaxis trial lasting 3 months
Participants	Canadian adults. 407 active and 411 placebo recipients. Recruitment specified cold proneness in the winter months.
Interventions	vit C 1g daily throughout study and 4g daily for first three days of respiratory illness compared with placebo
Outcomes	Incidence Fig 1, Duration Fig 2 and Severity, Fig 3.
Notes	Jadad 5<br>PD=I
Allocation concealment	A
**Study**	**Anderson 1974a**
Methods	Double blind trial randomised lasting three months. Four prophylaxis, two treatment and two placebo arms. This entry reports a prophylaxis trial
Participants	Canadian adults of both sexes. Data for this arm includes 277 vit C and 285 placebo.
Interventions	Vit C 1g daily and 4g at onset of illness versus placebo
Outcomes	Incidence Fig 1, Duration Fig 2 and Severity, Fig 3
Notes	Jadad 5 .<br>PD=I<br>Problems with one of placebo groups (#6) described in text
Allocation concealment	A
**Study**	**Anderson 1974b**
Methods	As for Anderson 1974a This arm a prophylaxis arm
Participants	Adults of both sexes 275 in the active arm and 285 in the placebo arm.
Interventions	1g of vit C daily versus placebo
Outcomes	Incidence Fig 1, Duration Fig 2 and Severity, Fig 3
Notes	Jadad 5.<br>PD=I
Allocation concealment	A
**Study**	**Anderson 1974c**
Methods	As for Anderson 1974a. This arm a prophylaxis arm

Participants	Adults of both sexes 308 in the active arm and 285 in the placebo arm
Interventions	2g of vit C daily versus placebo
Outcomes	Incidence Fig 1, Duration Fig 2 and Severity, Fig 3
Notes	Jadad 5 .<br>PD=I
Allocation concealment	A
**Study**	**Anderson 1974d**
Methods	As for Anderson 1974a This arm a prophylaxis arm
Participants	Adults of both sexes 331 in the active arm and 285 in the placebo arm
Interventions	0.25g of vit C daily versus placebo
Outcomes	Incidence Fig 1, Duration Fig 2 and Severity, Fig 3
Notes	Jadad 5.<br>PD=I
Allocation concealment	A
**Study**	**Anderson 1974e**
Methods	As for Anderson 1974a. This arm a therapeutic arm
Participants	Adults of both sexes. 275 in the active arm and 285 in the placebo arm
Interventions	4g vit C on first day of respiratory illness versus placebo
Outcomes	Duration Fig 4<br>Severity Fig 5
Notes	Jadad 5.<br>PD=I
Allocation concealment	A
**Study**	**Anderson 1974f**
Methods	As for Anderson 1974a. This arm a therapeutic arm
Participants	Adults of both sexes. 308 in the active arm and 285 in the placebo arm
Interventions	8g vit C on first day of respiratory illness versus placebo
Outcomes	Duration Fig 4<br>Severity Fig 5
Notes	Jadad 5.<br>PD=I
Allocation concealment	A
**Study**	**Anderson 1975a**
Methods	Double blind RCT. Design tests effects of vit C as therapy. Duration of study 15 weeks. Randomised double blind study with two active and one placebo arm. This arm used tablets as active agent.
Participants	Adults of both sexes 150 active and 146 placebo
Interventions	0.5 g weekly and 1.5 g on first day of illness with 1 g daily for next four days versus placebo.

Outcomes	Duration Fig 4 and Severity, Fig 5
Notes	Jadad 5<br>PD=I
Allocation concealment	A
**Study**	**Anderson 1975b**
Methods	As for Anderson 1975a. This arm used capsules as active agent
Participants	Adults of both sexes 152 active and 146 placebo
Interventions	0.5 g weekly and 1.5 g day 1 of illness with 1 g daily for next four days versus placebo.
Outcomes	Duration Fig 4 and Severity, Fig 5
Notes	Jadad 5<br>PD=I
Allocation concealment	A
**Study**	**Audera 2001a**
Methods	Randomized double-blind therapeutic therapeutic trial
Participants	Australian adults of both sexes 47 active and 42 placebo
Interventions	1g vit C for three days compared with placebo group who received 30 mg vit C daily for three days
Outcomes	Duration Fig 4 and Severity, Fig 5
Notes	Jadad 4<br>PD=I
Allocation concealment	A
**Study**	**Audera 2001b**
Methods	As for Audera 2001a
Participants	50 active and 42 placebo
Interventions	3g vit C for three days compared with placebo group who received 30 mg vit C daily for three days
Outcomes	Duration Fig 4 and Severity, Fig 5
Notes	Jadad 4 .<br>PD=I
Allocation concealment	A
**Study**	**Audera 2001c**
Methods	As for Audera 2001a
Participants	45 active and 42 placebo
Interventions	3g vit C with added flavonoids for three days compared with placebo group who received 30 mg vit C daily for three days
Outcomes	Duration Fig 4 and Severity, Fig 5
Notes	Jadad 4<br>PD=I

	As for Audera 2001
Allocation concealment	A
**Study**	**Bancalari 1984**
Methods	Double-blind, randomized prophylaxis trial. Duration 84 days
Participants	Healthy Chilean school children, male and female, aged 10 to 12 years. 32 active and 30 placebo
Interventions	2 g of vit C compared with placebo
Outcomes	Incidence Fig 1, Duration Fig 2
Notes	Jadad 3<br>PD=I
Allocation concealment	B
**Study**	**Briggs 1984**
Methods	Double blind randomised prophylaxis trial which ran over eight winters for one winter period of three or six months of commitment by each volunteer,
Participants	Australian healthy adults, male and female. 265 high dose recipients versus 263 low dose "placebo"
Interventions	1g of ascorbic acid plus 4g daily when respiratory symptoms occurred versus 50 mgs daily plus 200 mgs daily while symptoms lasted.
Outcomes	Incidence Fig 1, Duration Fig 2.
Notes	Jadad 3<br>PD=I<br>SD for duration was not published and it was estimated in the current review as SD=mean.
Allocation concealment	A
**Study**	**Carr 1981a**
Methods	Double blind identical twin prophylaxis study involving two groups of twins one group of which were living together and the other living apart. Carr 1981a deals with those living together. Duration 100 days
Participants	Australian males and females age range 14-64 years (mean 25 years) 51 pairs living together
Interventions	1G daily plus a multi vitamin tablet that contained 70 mgs vit C daily in each group, versus placebo.
Outcomes	Duration Fig 2 and Severity, Fig 3.
Notes	Jadad 4<br>PD=I<br>SD for duration was not published and the SD for the current review was calculated from the p value.
Allocation concealment	A
**Study**	**Carr 1981b**
Methods	As for Carr 1981, this report refers to the identical twins who lived apart,
Participants	Australian males and females age range 14-64 years (mean 25 years) 44

	identical twin pairs living apart.
Interventions	As for Carr 1981
Outcomes	Duration Fig 2 and Severity, Fig 3.
Notes	Jadad 4 .<br>PD=I<br>SD for duration was not published and the SD for the current review was calculated from the p value.
Allocation concealment	A
**Study**	**Carson 1975**
Methods	Double blind controlled prophylaxis trial Forty days duration.
Participants	UK healthy adults 121 vit C and 123 placebo
Interventions	1g vit C daily vs placebo
Outcomes	Incidence Fig 1
Notes	Jadad 3<br>PD=?
Allocation concealment	C
**Study**	**Charleston 1972**
Methods	Controlled prophylaxis trial. Single blind not randomised. Duration 15 weeks
Participants	Staff and students of The University of Strathclyde. UK. 47 active arm and 43 placebo arm participants.
Interventions	1g of vit C versus placebo. 1g vit C daily versus placebo
Outcomes	Incidence Fig 1, Duration Fig 2
Notes	Jadad 0<br>PD=?
Allocation concealment	C
**Study**	**Clegg 1975**
Methods	Apparently double blind randomised prophylaxis trial. 15 weeks duration
Participants	Healthy Scottish students 67 active and 70 placebo .
Interventions	1g vit C daily versus indistinguishable placebo.
Outcomes	Incidence Fig 1, Duration Fig 2
Notes	Jadad 2.<br>PD=I
Allocation concealment	B
**Study**	**Coulehan 1974a**
Methods	Double blind prophylaxis trial. Alternate allocation. Duration 14 weeks
Participants	USA. Residential students at a Navaho Indian school 131 active and 128 placebo .
Interventions	2g of vit C or placebo daily or placebo.

Outcomes	Incidence Fig 1, Duration Fig 2
Notes	Jadad 4.<br>PD=I<br>SD for duration was not published and it was estimated in the current review as SD=mean.
Allocation concealment	C
**Study**	**Coulehan 1974b**
Methods	See Coulehan 1974
Participants	Younger residential children. 190 active and 192 placebo
Interventions	1g vit C or placebo daily
Outcomes	Incidence Fig 1, Duration Fig 2
Notes	Jadad 4.<br>PD=I<br>SD for duration was not published and it was estimated in the current review as SD=mean.
Allocation concealment	C
**Study**	**Coulehan 1976**
Methods	Randomised double blind prophylaxis trial Duration 18 weeks in one school and 15 weeks in the other.
Participants	USA Children at two Navaho Indian residential schools aged 6-15 years. Both sexes. 428 active and 428 placebo
Interventions	1g vit C or placebo daily
Outcomes	Incidence Fig 1, Duration Fig 2
Notes	Jadad 4.<br>PD=I<br>SD for duration was not published and it was estimated in the current review as SD=mean.
Allocation concealment	A
**Study**	**Cowan 1942**
Methods	Controlled prophylaxis trial
Participants	US College students 208 active 155 placebo
Interventions	200 mg of vitamin C or placebo
Outcomes	Incidence Fig 1
Notes	Jadad 2<br>PD=?<br>SD for duration was not published and it was estimated in the current review as SD=mean.
Allocation concealment	B
**Study**	**Cowan 1950**
Methods	Randomised probably double blind therapeutic trial

Participants	US College students. 76 vit C and 77 placebo treated colds
Interventions	6g vitamin C versus placebo during the first 48 hours of symptoms
Outcomes	Duration Fig 4
Notes	Jadad 3<br>PD=?
Allocation concealment	B
**Study**	**Dahlberg 1944**
Methods	Controlled prophylaxis trial
Participants	Swedish army 1940. 1259 vit C 1266 placebo
Interventions	200 mg of vit C daily during the first 24 days of the 57 day study and 50 milligrams during the remainder versus placebo
Outcomes	Incidence Fig 1
Notes	Jadad 3<br>PD=?
Allocation concealment	B
**Study**	**Dick 1990**
Methods	Brief abstract report of three experimental prophylaxis studies using intense exposure to infected volunteers
Participants	24 Vit C and 24 placebo adult volunteers USA
Interventions	2G vit C daily versus placebo
Outcomes	Shown in Table 1. Not included in meta-analyses
Notes	Jadad 2<br>PD=?.<br>First of the three trials was reported under Mink 1988 in the earlier edition of this review.
Allocation concealment	B
**Study**	**Elwood 1976**
Methods	Double blind randomised prophylaxis trial
Participants	Wales Young mothers 339 vit C Vitamin C 349 placebo
Interventions	1g vit C daily versus placebo.
Outcomes	Incidence Fig 1. Duration Fig 2
Notes	Jadad 2<br>PD=?
Allocation concealment	B
**Study**	**Elwood 1977**
Methods	Double-blind randomized therapeutic trial Colds were classified either as simple or chest colds.
Participants	145 colds treated with vit C and 119 treated with placebo
Interventions	4g vit C daily for first 2.5 days of illness or placebo

Outcomes	Duration Fig 4
Notes	Jadad 2<br>PD=?<br>If the chest colds lasting more than 20 days are included in the comparison the statistically significant difference favouring vit C disappears.
Allocation concealment	B
**Study**	**Franz 1956**
Methods	Single blind prophylaxis study
Participants	German medical students and nurses 44 vit C plus or minus bioflavonoids and 45 who received placebo or bioflavonoids alone.
Interventions	205 mg vit C daily versus placebo.
Outcomes	Incidence Fig 1
Notes	Jadad 4<br>PD=?
Allocation concealment	A
**Study**	**Himmelstein 1998a**
Methods	Double blind randomised prophylaxis trial Duration two months prior to and one month following marathon race
Participants	U S Marathon runners<br>30 vit C and 14 placebo runners
Interventions	1 gram of vitamin C daily or placebo
Outcomes	Incidence Fig 1, Duration Fig 2 , Severity Fig 3
Notes	Jadad 3<br>PD=I<br>High and statistically significant differential dropout of placebo recipients (see text)
Allocation concealment	A
**Study**	**Himmelstein 1998b**
Methods	As for Himmelstein 1998b Sedentary controls of the marathon runners
Participants	US sedentary controls for marathon runners 23 vitamin and 25 placebo
Interventions	As for Himmelstein 1998
Outcomes	Incidence Fig 1, Duration Fig 2 , Severity Fig 3
Notes	Jadad 3<br>PD=I
Allocation concealment	A
**Study**	**Karlowski 1975a**
Methods	"Double" blind randomised four armed prophylaxis and therapeutic study nine months duration.<br>Three different arms were compared with one placebo arm<br>This arm prophylaxis

Participants	This arm 44 vit C recipients versus 46 placebo
Interventions	This prophylaxis arm 3g vit C daily versus placebo
Outcomes	Duration Fig 2
Notes	Jadad 4<br>PD=?<br>The authors believed that the benefits observed were attributable to the breaking of the patient blind but see Hemilä 1996a
Allocation concealment	B
**Study**	**Karlowski 1975b**
Methods	See Karlowski 1975a. This arm prophylaxis plus therapeutic load
Participants	57 vit C versus 46 placebo
Interventions	3g vit C and 3g supplementation when cold symptoms occurred
Outcomes	Duration Fig 2
Notes	Jadad 4.<br>PD=?<br>See Karlowski 1975
Allocation concealment	B
**Study**	**Karlowski 1975c**
Methods	See Karlowski 1975a This arm therapeutic only
Participants	43 vit C versus 46 placebo
Interventions	3g therapeutic dose vit C at time of onset of cold only
Outcomes	Duration Fig 4
Notes	Jadad 4<br>PD=?<br>See Karlowski 1975
Allocation concealment	B
**Study**	**Liljefors 1972**
Methods	Double-blind randomized crossover prophylaxis trial duration four weeks. In the first two weeks 25 participants received Vit C and 18 received placebo. As participants became ill they were removed from the trial and three personnel also withdrew from the crossover arm of the trial. In the second period, 18 received placebo and eight received vit C
Participants	40 Swedish army males who received altogether 33 two week courses of Vitamin C and 33 two week courses of placebo
Interventions	2 g vit C daily for two weeks and an identical placebo for the same period; crossover design
Outcomes	Incidence Fig 1
Notes	Jadad 3<br>PD=?
Allocation concealment	A

**Study**	**Ludvigsson 1977a**
Methods	Pilot double blind randomised prophylaxis study<br>Duration seven weeks
Participants	Sweden healthy schoolchildren, 80 Vit C and 78 placebo
Interventions	1g vit C vs placebo containing 30mg vit C
Outcomes	Incidence Fig 1, Duration Fig 2, Severity Fig 3
Notes	Jadad 3<br>PD=I<br>Pilot study to Ludviggson 1977b
Allocation concealment	B
**Study**	**Ludvigsson 1977b**
Methods	Double blind, randomised prophylaxis study<br>Duration three months
Participants	304 vit C 311 placebo
Interventions	1 G vit C versus placebo that contained 10 mg of vit C
Outcomes	Incidence Fig 1, Duration Fig 2, Severity Fig 3
Notes	Jadad 3<br>PD=I
Allocation concealment	B
**Study**	**Miller 1977a**
Methods	Double blind randomised prophylaxis identical twin study
Participants	12 pairs of identical twin children
Interventions	1G vit C daily versus placebo containing 50 mg vit C daily
Outcomes	Duration Fig 2<br>Severity Fig 3
Notes	Jadad 4.<br>PD=I
Allocation concealment	B
**Study**	**Miller 1977b**
Methods	Double blind randomised prophylaxis identical twin study
Participants	12 pairs of identical twin children
Interventions	750mg vit C daily or placebo containing 50 mg vit C
Outcomes	Duration Fig 2<br>Severity Fig 3
Notes	Jadad 4<br>PD=I
Allocation concealment	B
**Study**	**Miller 1977c**
Methods	Double blind randomised prophylaxis identical twin study

Participants	20 younger pairs of identical twin children
Interventions	500 mg vit C daily or placebo<br>containing 50 mg vit C
Outcomes	Duration Fig 2<br>Severity Fig 3
Notes	Jadad 4<br>PD=I
Allocation concealment	B
**Study**	**Moolla 1996a**
Methods	Double blind randomised prophylaxis trial of ultra marathon runners
Participants	South African Ultra marathon runners 13 vit C 19 placebo
Interventions	250 mg vit C or placebo administered for six weeks before the marathon and two weeks after marathon event
Outcomes	Incidence Fig 1
Notes	Jadad =3 PD= ? 1 of the four who reported respiratory symptoms in vit C recipients and 8 of 13 in placebo recipients reported that their respiratory symptoms were severe
Allocation concealment	B
**Study**	**Moolla 1996b**
Methods	Double blind randomised prophylaxis trial of controls for runners described in Moola 1996
Participants	Controls for runners in Moolla 1996a 11 vit C and 19 placebo
Interventions	250 mg vit C or placebo administered for six weeks before the marathon and two weeks afterwards
Outcomes	Incidence Fig 1
Notes	Jadad = 3 PD= ? 0 of the six who reported respiratory symptoms in vit C recipients and 4 of 7 in placebo recipients reported that their respiratory symptoms were severe
Allocation concealment	B
**Study**	**Peters 1993a**
Methods	Double blind randomised prophylaxis trial. Duration three weeks before and two weeks after ultra marathon.
Participants	South African ultra marathon runners 43 vitamin C and 41 placebo
Interventions	600 mg vit C versus placebo
Outcomes	Incidence Fig 1,<br>Duration Fig 2
Notes	Jadad 2<br>PD=I.
Allocation concealment	C
**Study**	**Peters 1993b**

Methods	As for Peters 1993. Non running controls for the marathon runners
Participants	Non-running control subjects 34 vitamin C and 39 placebo
Interventions	As for Peters 1993a
Outcomes	Incidence Fig 1<br>Duration Fig 2
Notes	Jadad 2.<br>PD=I
Allocation concealment	C
**Study**	**Peters 1996a**
Methods	Double blind randomised prophylaxis trial
Participants	South Africa Ultra marathon runners<br>44 vitamin C and 47 placebo
Interventions	0.5 g Vitamin C daily versus placebo 21 days prior to the race
Outcomes	Incidence Fig 1 Duration Fig 2
Notes	Jadad 3<br>PD=?<br>SD for duration was not published and it was estimated in the current review as SD=mean.
Allocation concealment	B
**Study**	**Peters 1996b**
Methods	As for Peters 1966a family controls of ultra marathon runners
Participants	41 vitamin C and 45 placebo in the family based controls
Interventions	As for Peters 1996a
Outcomes	Incidence Fig 1 Duration Fig 2
Notes	Jadad 3<br>PD=?<br>SD for duration was not published and it was estimated in the current review as SD=mean.
Allocation concealment	B
**Study**	**Pitt 1979**
Methods	Double blind, randomised prophylaxis trial. Eight weeks duration
Participants	USA Marine recruits, 331 vit C and 343 placebo recipients
Interventions	2g Ascorbic acid daily versus placebo
Outcomes	Incidence Fig 1, Duration Fig 2<br>Severity Figure 3
Notes	Jadad 5.<br>PD=I<br>SD for duration was not published and it was estimated in the current review as SD=mean.
Allocation concealment	A

**Study**	**Ritzel 1961**
Methods	Double blind randomised prophylaxis study duration two weeks
Participants	Children attending ski school in Switzerland 139 vit C, 140 placebo.
Interventions	1 g of vit C daily versus placebo
Outcomes	Incidence Fig 1, Duration Fig 2
Notes	Jadad 3<br>PD=I<br>SD for duration was not published and the SD for the current review was calculated from the P value.
Allocation concealment	A
**Study**	**Sabiston 1974**
Methods	Double blind randomized prophylaxis trial; duration a few weeks
Participants	Canadian male military recruits during winter subarctic exercises
Interventions	1g vit C daily or placebo for the duration of the exercise which is not specified.
Outcomes	Incidence Fig 1, Severity Fig 3
Notes	Jadad 1.<br>PD=I
Allocation concealment	C
**Study**	**Schwartz 1973**
Methods	Double blind experimental prophylaxis study with nasal instillation of virus after two weeks of pretreatment
Participants	Male US prison volunteers 11 vit C and 10 placebo
Interventions	3g vit C versus placebo which is not described.
Outcomes	Shown in Table 1. Not included in meta-analyses
Notes	Jadad 2 .<br>PD=?
Allocation concealment	C
**Study**	**Tyrrell 1977**
Methods	Randomised double blind therapeutic trial
Participants	UK Males and females 274 episodes treated with Vit C versus 329 treated with placebo
Interventions	4g vit C daily vs placebo for first 2.5 days of cold symptoms
Outcomes	Duration Fig 4, Severity Fig 5
Notes	Jadad 4.<br>PD=I
Allocation concealment	B
**Study**	**Van Straten 2002**
Methods	Double-blind randomized prophylaxis trial using specific form of vitamin C

	(Esther-C ascorbate, a natural form of vitamin C that "allows cells to efficiently absorb and retain high levels of vitamin".) Duration 60 days .
Participants	UK volunteers both sexes 84 vit C 84 placebo
Interventions	1g daily versus placebo
Outcomes	Incidence Fig 1, Duration Fig 2
Notes	Jadad 4.<br>PD=?
Allocation concealment	A
**Study**	**Walker 1967**
Methods	Experimental prophylaxis study in which healthy volunteers were intranasally inoculated with viruses.
Participants	UK adults both sexes. 47 vit C, 44 placebo,
Interventions	3g vit C versus placebo for 3 days before and six days after nasal instillation of virus.
Outcomes	Shown in Table 1. Not included in meta-analyses
Notes	Jadad 0.<br>PD=I
Allocation concealment	C
**Study**	**Wilson 1973a**
Methods	Double blind randomised prophylaxis study nine months duration
Participants	UK boarding school girls 70 vit C 58 placebo
Interventions	200 mg daily versus placebo
Outcomes	Duration Fig 2<br>Severity Fig 3
Notes	Jadad 4.<br>PD=?<br>Unique classification system makes comparison with other studies difficult.
Allocation concealment	A
**Study**	**Wilson 1973b**
Methods	As for Wilson 1973a
Participants	UK boarding school boys vit C 88, placebo 86
Interventions	As for Wilson 1973
Outcomes	Duration Fig 2<br>Severity Fig 3
Notes	Jadad 4.<br>PD=?<br>As for Wilson 1973
Allocation concealment	A

Characteristics of excluded studies

Study	Reason for exclusion
Asfora 1977	This Brazilian study involving 134 adults, which sought to evaluate the therapeutic benefits of use of 6 grams of vitamin C daily for five days following the onset of cold symptoms did not report comparisons between placebo and vitamin C but between vitamin C and the use of other drugs. No useful data for this review could be extracted. The paper revealed a strong bias of the investigator towards the therapeutic benefits of vitamin C. The Jadad score was 1.
Baird 1979	362 healthy volunteers aged 17 to 25 years were studied for 72 days in a trial of prophylaxis using a daily drink that contained either synthetic orange juice without ascorbic acid, synthetic juice with 80 milligrams of ascorbic acid added, or natural orange juice with 80 milligrams of ascorbic acid added. Daily records of symptoms were collected. There was a 14 to 21 percent reduction in total symptoms due to the common cold in the supplemented groups that was statistically significant (p less than 0.05). However the authors concluded that the clinical usefulness of the result did not support prophylactic ascorbic acid supplements in the well nourished adult. The study achieved a Jadad score of two and was well conducted. It was ruled ineligible for this review on the basis of the low dosage used.
Barnes 1961	A multivitamin preparation that included 200 milligrams of vitamin C was given to 23 members of a basketball team for seven weeks and the cold outcomes were compared with those of 16 other boys and girls of the same age and background. The basketballers included 13 girls and 10 boys who received their medication from the coaches. The controls included eight boys and eight girls who reported to the coaches daily. Days sick from cold were counted in each group The study took place over eight weeks during which the basketballers took medication on an average of 43 days. The active preparation was a multivitamin tablet that included 200 mg ascorbic acid incorporated in a multivitamin tablet daily for the basketballers and no treatment for the controls. Vitamin C n=23 Controls n=16. The only usable outcome was Mean days per person vitamin C 1.48 SD 2.65. Controls 6.87 SD 8.57 . However the study failed to meet our criteria. There was no semblance of blindness nor randomized allocation and no placebo medication was used. There were serious doubts about the comparability of the controls who were apparently not basketballers
Bendel 1955	120 children at a summer camp for two weeks were given 200 mg Vit C daily and their cold experience compared with that of participants in an earlier camp. No placebo group.
Bergquist 1943	A study involving supplementation with vitamin C of only 30 mg per day
Bessel-Lorck 1958	Descriptive cohort study of Berlin high school skiers. No placebo comparison.
Boines 1956	Descriptive cohort study of poliomyelitis sufferers. No placebo comparisons.
Brown 1945	Randomised controlled comparison of college age students. Outcome was "Colds that did not develop." No data that could be used in our meta-analyses, though benefit was claimed.
Chavance 1993	Randomised double blind controlled trial of 90 mg ascorbic acid daily in elderly participants. Excluded on the basis of dose. No benefit was demonstrated
Cuendet 1946	200 children in three mountain parishes took vitamin C supplements up to 300mg daily. There was no placebo control group .

Dyllick 1967	Cohort workplace study involving 200 recipients of 1g daily of Vitamin C whose respiratory experience was compared with that of those not receiving vit C. No placebo.
Elliot 1973	70 crew members of a Polaris submarine participated in a ten week well conducted randomized trial Jadad 3. Incidence of cold episodes was reported similar in the two groups but days or morbidity were said to be significantly less for sore throats and productive cough. However only the percentage difference between the vitamin C and placebo groups was published and the data cannot be included in our tables.
Fogelholm 1998	This Finnish study involved 75 athletes in a randomized trial of either vitamin C 1 gram daily together with vitamin E 294 mg daily and ubinquone 90 mg daily vs. an undescribed placebo. Methodologically strong study but was excluded from the meta-analyses because there were three antioxidants in the active preparation which were each hypothesized to be potentially beneficial, not just ascorbic acid.
Glazebrook 1942	Cohort study involving 1500 youth residents in an institution. Vitamin C was administered in milk to a group of 335 of the residents Dosage uncertain and inconsistent but apparently less than 200 mg per day. Comparisons with un supplemented group suggested some benefits to the supplemented group. The study was rejected on the basis of dosage.
Gormley 1977	Fourteen males of 29 members of a one year Antarctic expedition agreed to take vitamin C, 1 gram daily throughout their stay. and their health outcomes were compared with the remaining group who did not to take vitamin C. No difference in health was observed between the two groups. Excluded as no placebo comparison.
Gorton 1999	A technical training facility in Chile was the site of this cohort study in which the experience of 250 trainees who were given 3 grams a day of vitamin C during their ten-day course, was compared with a control group of 463 students who had been monitored in a similar way during the previous year. Excluded as no placebo group though authors claimed benefit from use of the vitamin C.
Hopfengartner 1944	Long term hospital baby study in which supplementation of 50 mg of vitamin C was used. Excluded on basis of dose.
Hunt 1994	57 elderly patients suffering from acute bronchitis or pneumonia who were being admitted to hospital for treatment, were randomized to receive, in addition to their other treatment, 200 mg of vitamin C per day or placebo. Excluded because the common cold was not the subject of interest.
Koytchev 2003	Four armed randomised double blind controlled trial involving 1167 participants, treated for their colds with 900 mg vitamin C daily plus or minus antihistamine and antipyretics. No placebo group to compare with the Vitamin C..
Masek 1974	Two large studies of Czeck coal miners comparing daily dose of 100mg vitamin C and placebo over a period of four or eight weeks. Excluded both on the basis of low dose of vitamin C used and inadequacy of data for inclusion in meta-analyses. The trials were neither randomised nor blind. Authors claimed benefits to the active recipients.
Niemi 1951	In this Finnish study, 1036 patients were observed during a three-month period and 516 of them were given 100 mg of vitamin C daily in addition to their usual diet. Excluded as no placebo was used and also low dose . No benefits claimed
Peters 1940	Short term baby supplementation study. No placebo comparison
RCGP Group	This controlled clinical therapeutic trial involved 270 family members of 78

1968	English general practitioners in winter 1967. 3G daily of Vitamin C was used to treat 147 active patients and 133 placebo recipients. Clinical scores for a range of symptoms were computed and stated not to be different between the two groups. However, raw data are not included and no usable data could be extracted from the paper.
Scheunert 1949	Large study involving factory workers in Germany between November 1942 in June 1943. Pills were distributed by foremen and managers in doses of 20, 50, 100 and 300 mg daily. A number of health outcomes were compared between ten different groups but these outcomes were not pertinent to this review .
Tebrock 1956	2000 adult subjects presenting with colds to industrial clinics were sequentially assigned to receive 200 mg daily of vitamin C, vitamin C plus flavonoids, flavonoids alone or placebo alone. All cases were again examined three days later by one of three physicians. The authors' conclusion from the extensively detailed tabulations is that "the overwhelming impression gained from the study is the singular lack of effect in altering the course of the common cold by either the bioflavonoids or the ascorbic acid". Recorded outcomes could not be used in this overview.

REFERENCES

References to studies included in this review

Anderson 1972{published data only}
Anderson T W, Reid DBW, Beaton GH. Vitamin C and the common cold: a double-blind trial. Canadian Medical Association Journal 1972;107:503-8.

Anderson 1974a{published data only}
Anderson TW, Suranyi G, Beaton GH. The effect on winter illness of large doses of vitamin C. Canadian Medical Association Journal 1974;111:31-6.

Anderson 1974b{published data only}
Anderson TW, Suranyi G, Beaton GH. The effect on winter illness of large doses of vitamin C. Canadian Medical Association Journal 1974;111:31-6.

Anderson 1974c{published data only}
Anderson TW, Suranyi G, Beaton GH. The effect on winter illness of large doses of vitamin C. Canadian Medical Association Journal 1974;111:31-6.

Anderson 1974d{published data only}
Anderson TW, Suranyi G, Beaton GH. The effect on winter illness of large doses of vitamin C. Canadian Medical Association Journal 1974;111:31-6.

Anderson 1974e{published data only}
Anderson TW, Suranyi G, Beaton GH. The effect on winter illness of large doses of vitamin C. Canadian Medical Association Journal 1974;111:31-6.

Anderson 1974f{published data only}
Anderson TW, Suranyi G, Beaton GH. The effect on winter illness of large doses of vitamin C. Canadian Medical Association Journal 1974;111:31-6.

Anderson 1975a{published data only}
Anderson TW, Beaton GH, Corey P, Spero L. Winter illness and vitamin C: the effect of relatively low doses. Canadian Medical Association Journal 1975;112(7):823-6.

Anderson 1975b{published data only}
Anderson TW, Beaton GH, Corey P, Spero L. Winter illness and vitamin C: the effect of relatively low doses. Canadian Medical Association Journal 1975;112(7):823-6.

Audera 2001a{published data only}

Audera C, Patulny RV, Sander BH, Douglas RM. Mega-dose vitamin C in treatment of the common cold: a randomised controlled trial. Medical Journal of Australia 2001;175(7):359-62.

Audera 2001b {published data only}
Audera C, Patulny RV, Sander BH, Douglas RM. Mega-dose vitamin C in treatment of the common cold: a randomised controlled trial. Medical Journal of Australia 2001;175(7):359-62.

Audera 2001c {published data only}
Audera C, Patulny RV, Sander BH, Douglas RM. Mega-dose vitamin C in treatment of the common cold: a randomised controlled trial. Medical Journal of Australia 2001;175(7):359-62.

Bancalari 1984 {published data only}
Bancalari M, Seguel C, Flor NC, Inez RA, Carlos CM. The value of vitamin C in the prevention of acute respiratory infections in schoolchildren [English translation available from harri.hemila@helsinki.fi] [Valor Profilactico de la Vitamina C en infeccciones Pespiratorias Agudas del escolar]. Revista Medica de Chile 1984;112:871-6.

Briggs 1984 {published data only}
Briggs M. Vitamin C and Infectious Disease: A Review of the Literature and the Results of a Randomised Double Blind Prospective Study Over Eight Years. In: Briggs MH, editor(s). Recent Vitamin Research. Bocaraton: CRC Press, 1984:39-82.

Carr 1981a {published data only}
Carr AB, Einstein R, Lai LY, Martin NG, Starmer GA. Vitamin C and the common cold: a second MZ Cotwin control study. Acta Geneticae Medicae et Gemellologiae (Roma) 1981;30(4):249-55.

Carr 1981b {published data only}
Carr AB, Einstein R, Lai LY, Martin NG, Starmer GA. Vitamin C and the common cold: a second MZ Cotwin control study. Acta Geneticae Medicae et Gemellologiae (Roma) 1981;30(4):249-55.

Carson 1975 {published data only}
Carson M, Cox H, Corbett M, Pollitt N. Vitamin C and the common cold. Journal of the Society of Occupational Medicine 1975;25(3):99-102.

Charleston 1972 {published data only}
Charleston SS, Clegg KM. Ascorbic acid and the common cold[see also Acta Vitminologica Enzymologica 1974;28:101-2]. Lancet 1972;1(765):1401-2.

Clegg 1975 {published data only}
Clegg KM, Macdonald JM. L-Ascorbic acid and D-isoascorbic acid in a common cold survey. American Journal of Clinical Nutrition 1975;28(9):973-6.

Coulehan 1974a {published data only}
Coulehan JL, Reisinger KS, Rogers KD, Bradley DW. Vitamin C prophylaxis in a boarding school. New England Journal of Medicine 1974;290(1):6-10.

Coulehan 1974b {published data only}
Coulehan JL, Reisinger KS, Rogers KD, Bradley DW. Vitamin C prophylaxis in a boarding school. New England Journal of Medicine 1974;290(1):6-10.

Coulehan 1976 {published data only}
Coulehan JL, Eberhard S, Kapner L, Taylor F, Rogers K, Garry P. Vitamin C and acute illness in Navajo school children. New England Journal of Medicine 1976;295(18):973-7.

Cowan 1942 {published data only}
Cowan D, Diehl HS, Baker AB. Vitamins for the prevention of colds. JAMA 1942;120:1268-71.

Cowan 1950 {published data only}
Cowan DW, Diehl HS. Anithistaminic agents and ascorbic acid in the early treatment of the common cold. JAMA 1950;143:421-4.

Dahlberg 1944 {published data only}
Dahlberg G, Engel A, Rydin H. The value of ascorbic acid as a prophylactic against common colds. Acta Medica Scandinavica 1944;119:540-61.

Dick 1990 {published data only}
Dick EC, Mink KA, Olander D, Schult PA, Jennings LC, Inhorn SL. Amelioration of rhinovirus type 16 (RV 16) colds in ascorbic acid supplemented volunteers. 30th ICAC Proceedings. 1990.

Elwood 1976 {published data only}

Elwood PC, Lee HP, St LA, Baird M, Howard AN. A randomized controlled trial of vitamin C in the prevention and amelioration of the common cold. British Journal of Preventive & Social Medicine 1976;30(3):193-6.

Elwood 1977{published data only}
Elwood P, Hughes SJ, Leger AS. A randomized controlled trial of the therapeutic effect of vitamin C in the common cold. Practitioner 1977;218(1303):133-7.

Franz 1956{published data only}
Franz WL, Sands GW, Heyl HL, Hanover NH. Blood ascorbic acid level in bioflavonoid and ascorbic acid therapy of common cold. JAMA 1956;162:1224-6.

Himmelstein 1998a{published data only}
Himmelstein SA, Robergs RA, Koehler KM, Lewis SL, Qualls CR. Vitamin C supplementation and upper respiratory tract infections in marathon runners[see also Doctoral dissertation submitted to University of New Mexico Albuquerque 1996]. Journal of Exercise Physiology Online 1998;1(2):1-21.

Himmelstein 1998b{published data only}
Himmelstein SA, Robergs RA, Koehler KM, Lewis S. L., Qualls CR. Vitamin C supplementation and upper respiratory tract infections in marathon runners [see also Doctoral dissertation submitted to University of New Mexico Albuquerque 1996]. Journal of Exercise Physiology Online 1998;1(2):1-21.

Karlowski 1975a{published data only}
Karlowski TR, Chalmers TC, Frenkel LD, Kapikian AZ, Lewis TL, Lynch JM. Ascorbic acid for the common cold. A prophylactic and therapeutic trial [see also Ann NY Acad Sci 1975;258:505-12]. JAMA 1975;231(10):1038-42.

Karlowski 1975b{published data only}
Karlowski TR, Chalmers TC, Frenkel LD, Kapikian AZ, Lewis TL, Lynch JM. Ascorbic acid for the common cold. A prophylactic and therapeutic trial [see also Ann NY Acad Sci 1975;258:505-12]. JAMA 1975;231(10):1038-42.

Karlowski 1975c{published data only}
Karlowski TR, Chalmers TC, Frenkel LD, Kapikian AZ, Lewis TL, Lynch JM. Ascorbic acid for the common cold. A prophylactic and therapeutic trial [see also Ann NY Acad Sci 1975;258:505-12]. JAMA 1975;231(10):1038-42.

Liljefors 1972{published data only}
Liljefors I. Vitamin C and the common cold. Lakartidningen 1972;69(28):3304-5.

Ludvigsson 1977a{published data only}
Ludvigsson J, Hansson LO, Tibbling G. Vitamin C as a preventive medicine against common colds in children. Scandinavian Journal of Infectious Diseases 1977;9:91-8.

Ludvigsson 1977b{published data only}
Ludvigsson J, Hansson LO, Tibbling G. Vitamin C as a preventive medicine against common colds in children. Scandinavian Journal of Infectious Diseases 1977;9:91-8.

Miller 1977a{published data only}
Miller JZ, Nance WE, Norton JA, Wolen RL, Griffith RS, Rose RJ. Therapeutic effect of vitamin C. A co-twin control study. JAMA 1977;237(3):248-51.

Miller 1977b{published data only}
Miller JZ, Nance WE, Norton JA, Wolen RL, Griffith RS, Rose RJ. Therapeutic effect of vitamin C. A co-twin control study. JAMA 1977;237(3):248-51.

Miller 1977c{published data only}
Miller JZ, Nance WE, Norton JA, Wolen RL, Griffith RS, Rose RJ. Therapeutic effect of vitamin C. A co-twin control study. JAMA 1997;237(3):248-51.

Moolla 1996a{unpublished data only}
*Moolla ME. The effect of supplemental anti-oxidants onthe Incidence and Severity of upper respiratory infections in Ultra Marathon runners. Unpublished Master thesis. University of Capetown. Available on request from Dr L M Weight, weight@curie.uct.ac.za 1996.

Moolla 1996b{unpublished data only}
*Moolla ME. The effect of supplemental anti-oxidants on the Incidence and Severity of upper respiratory infections in Ultra Marathon runners. Unpublished Master thesis at The University of Capetown. Available from Dr L M Weight, weight@curie.uct.ac.za 1996.

Peters 1993a{published data only}

Peters EM, Goetzsche JM, Grobbelaar B, Noakes TD. Vitamin C supplementation reduces the incidence of post-race symptoms of upper-respiratory-tract infection in ultramarathon runners. American Journal of Clinical Nutrition 1993;57:170-4.

Peters 1993b{published data only}
Peters EM, Goetzsche JM, Grobbelaar B, Noakes TD. Vitamin C supplementation reduces the incidence of postrace symptoms of upper-respiratory-tract infection in ultramarathon runners. American Journal of Clinical Nutrition 1993;57:170-4.

Peters 1996a{published data only}
Peters EM, Goetzsche JM, Joseph LE, Noakes TD. Vitamin C as effective as combinations of antioxidant nutrients in reducing symptoms of upper respiratory tract infection in ultramarathon runners. South African Journal of Sports Medicine 1996;11:23-7.

Peters 1996b{published data only}
Peters EM, Goetzsche JM, Joseph LE, Noakes TD. Vitamin C as effective as combinations of antioxidant nutrients in reducing symptoms of upper respiratory tract infection in ultramarathon runners. South African Journal of Sports Medicine 1996;11:23-7.

Pitt 1979{published data only}
Pitt HA, Costrini AM. Vitamin C prophylaxis in marine recruits. JAMA 1979;241(9):908-11.

Ritzel 1961{published data only}
*Ritzel G. [English translation available from harri.hemila@helsinki.fi] [Kritische beurteilung des vitamins C als prophylacticum und therapeuticum der erkältungskrankheiten [see also JAMA 1976;235:1108]]. Helvetica Medica Acta 1961;28:63-8.

Sabiston 1974{published data only}
Sabiston BH,Radomski MW. Health Problems and Vitamin C in Canadian Northern Military operations. Defence Research Board DCIEM Report No 74-R-102. 1974.

Schwartz 1973{published data only}
Schwartz AR, Togo Y, Hornick RB, Tominaga S, Gleckman RA. Evaluation of the efficacy of ascorbic acid in prophylaxis of induced rhinovirus 44 infection in man. Journal of Infectious Diseases 1973;128(4):500-5.

Tyrrell 1977{published data only}
Tyrrell D, Craig JW, Meada TW, White T. A trial of ascorbic acid in the treatment of the common cold. British Journal of Preventive & Social Medicine 1977;31(3):189-91.

Van Straten 2002{published data only}
Van Straten M, Josling P. Preventing the common cold with a vitamin C supplement: a double-blind, placebo-controlled survey. Advances in Therapy 2002;19(3):151-9.

Walker 1967{published data only}
Walker G, Bynoe ML, Tyrrell DA. Trial of ascorbic acid in prevention of colds. British Medical Journal 1967;1(540):603-6.

Wilson 1973a{published data only}
Wilson C, Loh HS, Foster FG. Common cold symptomatology and vitamin C. European Journal of Clinical Pharmacology 1973;6(3):196-202.

Wilson 1973b{published data only}
Wilson C, Loh HS, Foster FG. Common cold symptomatology and vitamin C. European Journal of Clinical Pharmacology 1973;6(3):196-202.

References to studies excluded from this review

Asfora 1977
Asfora J. Vitamin C in high doses in the treatment of the common cold. Int J of Vitamin and Nutrition Research Supplement 1977;16:219-34.

Baird 1979
Baird IM, Hughes RE, Wilson HK, Davies JE, Howard AN. The effects of ascorbic acid and flavonoids on the occurrence of symptoms normally associated with the common cold. American Journal of Clinical Nutrition 1979;32:1686-90.

Barnes 1961
Barnes FE. Vitamin supplements and the incidence of colds in high school basketball players. North Carolina Medical Journal 1961;22:22-6.

Bendel 1955

*Bendel A. Redoxon als Prophylaktikum bei Erkältungskrankheiten. Therapeutische Umschau und Medizinische Bibliographie 1955;12:87-8.

Bergquist 1943
Bergquist G. Om Profylaktiska medel mot s.k. förkylningssjukdomar. Svenska Lakartidningen 1943;40:2042-7.

Bessel-Lorck 1958
Bessel-Lorck C. Prevention of cold in young subjects in ski camps [Erkal-tungsprophylaxe bei Jugendlichen im Skilager]. Medizinizche 1959;44:2126-7.

Boines 1956
Boines GJ, Horoschak S. Hesperidin and asc orbic acid in the prevention of upper respiratory infections. International Record of Medicine and General Practice Clinics 1956;169(2):73-6.

Brown 1945
Brown WMB, Mahoney F, Niedringhaus A, Locke A. Weather and susceptibility in relation to the spread of common cold; effect of ascorbic acid, in massive dosage, on duration. Journal of Immunology 1945;50:161-77.

Chavance 1993
Chavance M, Herbeth B, Lemoine A, Zhu BP. Does multivitamin supplementation prevent infections in healthy elderly subjects? A controlled trial. International Journal of Vitamin and Nutrition Research 1993;63:11-6.

Cuendet 1946
Cuendet O. Ernahrunga-und Vitaminfragen im Saas-Tal. Praxis 1946;17:378-82.

Dyllick 1967
Dyllick H. Werkarztliche erfahrungen mit vitamin C. Medizinische Welt 1967;17:1098-100.

Elliot 1973
Elliot B. Ascorbic acid: efficacy in the prevention of symptoms of respiratory infection on a polaris submarine. International Research Communications System: Respiratory System 1973;73(5):12.

Fogelholm 1998
Fogelholm M, Vasankari T,Borg P,Katila R, Tuomi T. Lack of effect of long-term antioxidant supplementation on incidence of upper respiratory tract infections in athletes. Proceedings of the Nutrition Society of London. ;571998:16A.

Glazebrook 1942
Glazebrook AJ, Thomson S. The administration of vitamin C in a large institution and its effect on general health and resistance to infection. Journal of Hygiene 1942;42:1-19.

Gormley 1977
Gormly PJ. Megadosage of ascorbic acid in an Antarctic expedition. British Jounal of Nutrition 1977;37(2):269-77.

Gorton 1999
Gorton H, Jarvis K. The effectiveness of vitamin C in preventing and relieving the symptoms of virus-induced respiratory infections. Journal of Manipulative and Physiological Therapeutics 1999;22(8):530-3.

Hopfengartner 1944
Hopfengärtner C. Vitaminprophylaxe bei Säuglingen. Monatsschrift fur Kinderheilkunde 1944;92:330-4.

Hunt 1994
Hunt CCN, Annan G, Habibzedeh N, Schorah CJ. The Clinical Effects of Vitamin C Supplementation in elderly Hospitalised Patients with Acute Respiratory Infections. International Journal of Vitamin and Nutrition Research 1994;64:212-9.

Koytchev 2003
Koytchev R, Vlahov V, Bacratcheva N, Giesel B, Gawronska-Szklarz. Evaluation of the efficacy of a combined formulation (Grippostad-C) in the therapy of symptoms of common cold: a randomized, double-blind, multicenter trial. EEvaluation of the efficacy of a combined formulation (Grippostad-C) in the therapy of symptoms of common cold: a randomized, double-blind, multicenter trial. International Journal of Clinical Pharmacology & Therapeutics 2003;41(3):114-25.

Masek 1974
Masek JHF, Neradilova M, Hejda S. The role of vitamin C in the treatment of acute infections of the upper respiratory pathways. Acta Vitaminologica et Enzymologica 1974;28(1-4):85-95.

Niemi 1951
Niemi TR. Acute respiratory Infections and Vitamin C. Duodecim 1951;67:360-8.

Peters 1940
Peters M. About the increase in resistance by daily prophylactic vitamin C medication [Zur Frage der resistenzsteigernden Wirkung prophylaktischer Dauermedikation von vitamin C]. Zeitschrift fur Kinderheilkunde 1940;61:179-83.

RCGP Group 1968
RCGP Group. General practitioner clinical trials: ineffectiveness of Vitamin C in treating coryza. The Practitioner 1968;200:442-5.

Scheunert 1949
Scheunert VA. Adult requirements for vitamin C [Der Tagesbedarf des Erwachsenen an Vitamin C]. Internationale Zeitschrift fur Vitaminforschung 1949;20:374-86.

Tebrock 1956
Tebrock HE, Arminio JJ, Ossining NY, Johnston JH. Usefulness of bioflavonoids and ascorbic acid in treatments of common cold. JAMA 1956;162:1227-33.

References to studies awaiting assessment

Bibile 1966
Bibile S. Vitamin C in the prophylaxis and treatment of the common cold. Proceedings of the Ceylon Association of Advanced Science 1966;1(9):7.

Additional references

Anderson 1977
Anderson T. Large scale studies with vitamin C. Acta Vitaminologica et Enzymologica 1977;31(1-5):43-50.

Chalmers 1975
Chalmers TC. Effects of Ascorbic Acid on the Common Cold. The American Journal of Medicine 1975;58:532-6.

Chalmers 1996
Chalmers TC. Dissent to the preceding article by H Hemila. Journal of Clinical Epidemiology 1996;49:1085.

Dickersin 1994
Dickersin K, Scherer R, Lefebvre C. Identifying relevant studies for systematic reviews. BMJ 1994;309:1286-91.

Dykes 1975
Dykes MH, Meier P. Ascorbic acid and the common cold. Evaluation of its efficacy and toxicity. JAMA 1975;231(10):1073-9.

Hemilä 1992
HemiläH. Vitamin C and the common cold. British Journal of Nutrition 1992;67:3-16.

Hemilä 1994
Hemilä H. Does vitamin C alleviate the symptoms of the common cold? - a review of current evidence. Scandinavian Journal of Infectious Diseases 1994;26:1-6.

Hemilä 1995
Hemilä H, Herman, Zelek S. Vitamin C and the common cold: a retrospective analysis of Chalmers' review. Journal of the American College of Nutrition 1995;14(2):116-23.

Hemilä 1996a
Hemilä H. Vitamin C, the placebo effect, and the common cold: a case study of how preconceptions influence the analysis of results. Journal of Clinical Epidemiology 1996;49(10):1079-84.

Hemilä 1996b
Hemilä H. Vitamin C and common cold incidence: a review of studies with subjects under heavy physical stress. International Journal of Sports Medicine 1996;17(5):379-83.

Hemilä 1996c
Hemilä H. Vitamin C supplementation and common cold symptoms: problems with inaccurate reviews. Nutrition 1996;12(11/12):804-9.

Hemilä 1996d
Hemilä H. Response to the Dissent by Thomas Chalmers. Journal of Clinical Epidemiology 1996;49:1087.

Hemilä 1997a
Hemilä H. Vitamin C intake and susceptibility to the common cold. British Journal of Nutrition 1997;77:59-72.

Hemilä 1997b
Hemilä H. Vitamin C supplementation and the common cold - was Linus Pauling right or wrong?. International Journal of Vitamin and Nutrition Research 1997;67:329-35.

Hemilä 1997c
Hemilä H. Vitamin C and Infectious diseases. In: Packer L, Fuchs J, editor(s). Vitamin C in Health and Disease. NY: Marcel Dekker, 1997:471-503.

Hemilä 1999a
Hemilä H, Douglas RM. Vitamin C and acute respiratory infections. International Journal of Tuberculosis and Lung Disease 1999;3(9):756-61.

Hemilä 1999b
Hemilä 1999. Vitamin C supplementation and common cold symptoms: factors affecting the magnitude of benefit. Medical Hypotheses 1999;52:171-8.

Hemilä 2004
Hemilä H. Vitamin C supplementation and respiratory infections in military recruits: a systematic review. Military Medicine 2004:in press.

Hrobartsson 2001
Hrobjartsson A, Gøtzsche PC. Is the placebo powerless? An analysis of clinical trials comparing placebo with no treatment. New England Journal of Medicine 2001;344:1594-602.

Jadad 1996
Jadad AR , Moore RA, Carroll D, Jenkinson C, Reynolds DJM, Gavaghan DJ et al. Assessing the quality of reports of randomized clinical trials: Is blinding necessary?. Controlled Clinical Trials 1996;17:1-12.

Kleijnan 1992
Kleijnen J, Knipschild P. The comprehensiveness of Medline and Embase computer searches. Pharmaceutisch Weekblad. Scientific edition 1992;14:316-20.

Kleijnen 1989
Kleijnen J, ter Riet G, Knipschild PG. Vitamin C and the common cold; review of a megadose of literature. Nederlands Tijdschrift voor Geneeskunde 1989;133:1532-35.

Pauling 1970a
Pauling L. Vitamin C and the Common Cold. San Francisco: WH Freeman and Co, 1970.

Pauling 1970b
Pauling L. Evolution and the need for ascorbic acid. Proceedings of the national Academy of Science USA 1970;67(4):1643-8.

Pauling 1971a
Pauling L. The significance of the evidence about ascorbic acid and the common cold. Proceedings of the National Academy of Science 1971;68(11):2678-81.

Pauling 1971b
Pauling L. Ascorbic acid and the common cold. American Journal of Clinical Nutrition 1971;24:1294-9.

Pauling 1976
Pauling L. Vitamin C, Common Cold, and the Flu. San Francisco: Freeman, 1976.

Wilson 1970
Wilson CWM, Loh HS. . Acta Allergologica 1970;24:367.

** Indicates the major publication for the study*

COMMENTS AND CRITICISMS

Unit of analysis issues

Summary:

Further to David Wooff's comment, I suspect there may be other statistical flaws in this review that could be placed under the heading, 'unit of analysis errors'.

At least one study (Lugviggson) appears to be a cluster randomized trial, yet there is no discussion of the possible over-weighting of this study when naively included in the meta-analyses.

At least two studies appear to be twin studies (Carr and Miller). Should the matching be taken into account in the analysis, in a similar way to a simple cross-over trial?

The particular meta-analysis for 'Mean symptom days per person' in the comparison 'Vitamin C 1G daily or more vs placebo' worries me considerably. Of the six studies (10 contributions) included in this analysis, I suspect that at most two are free of unit of analysis errors of various kinds. This makes it a wonderful teaching example, but for the wrong reasons.

I certify that I have no affiliations with or involvement in any organisation or entity with a direct financial interest in the subject matter of my criticisms.

Author's Reply:

Ludvigsson writes explicitly "Every class was divided at random into two groups." In our opinion this statement means that Ludvigsson was taking one class and he divided the subjects of that one class to two groups 'at random,' and then he went to another class and similarly randomized the second class. We disagree that cluster randomisation applied here.

As to the two small twin trials: Miller 1977 expicitly stated that "analysis of the paired comparisons…" so we conclude their SE values in their main table are based on paired t-test, event though this is not explicitly stated in their methods; Carr 1981 explicitly stated "the results for the six summary cold variables of the paired analyses of variance between active and placebo groups are shown…" so we conclude their P-values refer to paired analyses. In any case, the mean difference between the groups is the same whether we calculate difference of means or mean of paired differences. Failure to take into account the pairing of data would mean that we would be over-conservative in our estimate of the precision of any effect, but it is unlikely that this issue would anyway have influenced our conclusions in a meaningful way.

In the current review we have not used as an outcome variable mean symptom days per person but have concentrated on mean symptom days per episode.

Contributors:

Julian Higgins

COMMENTS AND CRITICISMS

Flaws in statistical analysis?

Summary:

There appear to be several instances where there is considerable overlap between studies, but they are treated as independent studies as far as the meta-analysis is concerned. For example, the Anderson 1974, 1974a, 1974b studies seem to be treated as independent in graph (comparison 01, outcome 04), but the control groups seem identical, and 275 people in the treatment group seem the same in each study. The effect is to inflate the value of this study. Indeed, the difference between the treatment groups for Anderson 1974a, 1974b (33 new people, *all* apparently with one or more respiratory episodes) raises further issues.

I certify that I have no affiliations with or involvement in any organisation or entity with a direct financial interest in the subject matter of my criticisms.

Author's Reply:

In the new edition of the review we have avoided this problem described above by combining all trial arms that were compared with the one placebo group into one trial arm for purposes of the meta-alysis

Contributors:
David Wooff

COMMENTS AND CRITICISMS

Doses too small

Summary:
One gram daily is a small dose. Most mammals make 3 or more grams in their livers. Any practitioner of orthomolecular medicine knows that a minimum of several grams a day is needed to surely prevent a cold, and as much as 20 grams to cure one in progress. Not one trial in your RCT's qualifies.

I certify that I have no affiliations with or involvement in any organisation or entity with a direct financial interest in the subject matter of my criticisms

Author's Reply:
The practitioners of orthomolecular medicine have not to our knowledge published any controlled trial evidence on which this comment is based. As we have said in the review, there is no reasonable doubt that vitamin C supplementation plays some biological role in defence, and there is tantalising evidence from the Anderson 1974 study that a single therapeutic dose of 8 grams at commencement of a cold may have had a useful therapeutic effect.

We believe there is a case for rigorous evaluation of the possibility that very large doses (of the order of 8g daily in adults for periods up to five days after the onset of symptoms) could produce benefits that were not seen at lower doses.

In view of the greater propensity of children to colds and the greater benefits observed in the child prophylaxis studies, they may be the group in which to explore this approach (with an appropriately pro-rated dose for weight). We add however a caution. Although studies in which doses of 1 or 2 g daily of vitamin C have been used for several months have not produced convincing evidence of adverse effects to the volunteers. Dosage of the kind discussed here needs to be carefully monitored for adverse effects especially in children.

Contributors:
Reuven Gilmore

GRAPHS

To view a graph or table, click on the outcome title of the summary table below.

To view graphs using MetaView, click the "Outline" button in the menu bar, and then select "Metaview graphs" from the Links.

01 Development of colds while on prophylaxis				
Outcome title	No. of studies	No. of participants	Statistical method	Effect size
01 Proportions developing	29	11077	Relative Risk	0.96

one or more cold episodes during prophylaxis			(Random) 95% CI	[0.92, 1.00]

02 Duration of colds developing on prophylaxis

Outcome title	No. of studies	No. of participants	Statistical method	Effect size
01 Mean symptom days per respiratory episode standardised against control group	30	9676	Weighted Mean Difference (Random) 95% CI	-9.73 [-14.07, -5.39]

03 Severity of colds developing on prophylaxis

Outcome title	No. of studies	No. of participants	Statistical method	Effect size
01 Indicators of severity of episodes experienced while on prophylaxis	15	7045	Standardised Mean Difference (Random) 95% CI	-0.13 [-0.21, -0.04]

04 Duration of colds treated with vitamin C or placebo

Outcome title	No. of studies	No. of participants	Statistical method	Effect size
01 Mean symptom days per episode standardised against control group	7	3294	Weighted Mean Difference (Random) 95% CI	-2.54 [-10.09, 5.02]

05 Severity of colds treated with vitamin C or placebo

Outcome title	No. of studies	No. of participants	Statistical method	Effect size
01 Indicators of severity of episodes for which vit C was used as therapy	4	2753	Standardised Mean Difference (Random) 95% CI	-0.07 [-0.16, 0.02]

COVER SHEET

Title	**Vitamin C for preventing and treating the common cold**
Reviewer(s)	**Douglas RM, Hemila H, D'Souza R, Chalker EB, Treacy B**
Contribution of reviewer(s)	**Bob Douglas (BD) conceived the review, screened retrieved papers against inclusion criteria, appraised the quality of papers, abstracted data, entered data into RevMan, analysed and interpreted the data, and wrote the review. He is the guarantor of the review.**<br><br>**Harri Hemila (HH) carefully reviewed drafts of the second edition of the review (2004), assisted in paper retrieval,**

proposed alterations to data presentation, checked data entries and contributed significant input to the text. He will take over responsibility for maintaining future editions of the review.

Ron D'Souza (RS) helped to assemble the update review database and shared with BD in the re-screening of all papers and their assessment of quality.

Elizabeth Chalker (EC) wrote the protocol of the first edition of the review developed the initial search strategy, undertook the searches, organised retrieval of papers, screened papers against inclusion criteria and appraised the quality of papers for that edtion

Barbara Treacy (BT) prepared overviews and summaries of published studies in preparation for the first version of the review (1998).

Issue protocol first published	Information not available
Issue review first published	1998/1
Date of most recent amendment	06 July 2004
Date of most recent SUBSTANTIVE amendment	10 August 2004
Most recent changes	This is the second edition of this review. The first edition covered the period from 1970 to 1997 and overviewed, using Cochrane methods, trials which had been previously studied by two other meta-analysts Kleijnen and Hemilä. For this edition, Hemilä who has been extensively involved in over-viewing this literature in the past fifteen years has joined the Cochrane review team and the review has been completely rewritten and includes studies from 1942 up to the present. 25 new trials have been added to the review. Of particular interest in this edition is the evidence presented of the effect of vitamin C on the common colds of marathon runners and others exposed to severe physical and cold stress. The review argues that vitamin C plays some biological role in defence against the common cold but that prophylaxis with high doses of vitamin C has no evidence based justification for members of the general population. It also points to the fact that most of the trials in which Vitamin C was used as therapy at onset of cold symptoms have revealed no benefit. It proposes further work on this aspect of the issue as there have been no therapeutic trials in children.
Date new studies	Information not supplied by reviewer

sought but none found	
Date new studies found but not yet included/excluded	Information not supplied by reviewer
Date new studies found and included/excluded	13 January 2004
Date reviewers' conclusions section amended	13 June 2004
Contact address	Em Prof Robert Douglas<br>Visiting Fellow<br>National Centre for Epidemiology and Population Health<br>The Australian National University<br>34 Nungara Place<br>Aranda<br>2614<br>ACT<br>AUSTRALIA<br>tel: +61 2 62533138<br>bobdouglas@netspeed.com.au<br>fax: +61 2 62533138
Cochrane Library number	CD000980
Editorial group	Cochrane Acute Respiratory Infections Group
Editorial group code	HM-ARI

SOURCES OF SUPPORT

External sources of support

- Commonwealth Department of Health and Ageing AUSTRALIA

Internal sources of support

- Australian National University AUSTRALIA

SYNOPSIS

Vitamin C in daily doses as high as 2g daily is not a panacea for either the prevention or treatment of the common cold

Evidence from trials of vitamin C tablets in the prevention and treatment of the common cold shows that with the exception of trials in people exposed to short periods of extreme physical and/or cold stress (including marathon runners and skiers), regular supplementation does not lower the probability of getting a cold. Regular supplementation is fairly consistently associated with minor reduction in duration, and sometimes in the severity of cold symptoms but this is of doubtful clinical usefulness. When high doses of vitamin C are taken at the onset of cold symptoms in an effort to treat colds, they have not been shown to reduce either the duration or severity of symptoms.

Index Terms

Medical Subject Headings (MeSH)
Administration, Oral; Ascorbic Acid [administration & dosage] [therapeutic use]; Common Cold [drug therapy] [prevention & control]; Respiratory Tract Infections [drug therapy] [prevention & control]

Mesh check words: Human

Copyright © 2004 The Cochrane Collaboration. Published by John Wiley & Sons, Ltd.
All rights reserved. No part of The Cochrane Library may be reproduced, stored in a retrieval system or transmitted in any form or by any means, electronic, mechanical, photocopying, recording, scanning or otherwise, except under the terms of the Copyright, Designs and Patents Act 1988 or under the terms of a licence issued by the Copyright Licensing Agency Ltd, 90 Tottenham Court Road, London W1T 4LP, UK, without the permission in writing of John Wiley & Sons, Ltd, The Atrium, Southern Gate, Chichester, West Sussex PO19 8SQ, UK, or emailed to permreq@wiley.co.uk, or faxed to (+44) 1243 770620.
